# Measurement invariance of the SF-12 among different demographic groups: The HELIUS study

**DOI:** 10.1371/journal.pone.0203483

**Published:** 2018-09-13

**Authors:** Henrike Galenkamp, Karien Stronks, Lidwine B. Mokkink, Eske M. Derks

**Affiliations:** 1 Department of Public Health, Amsterdam Public Health Research Institute, Amsterdam UMC, University of Amsterdam, Amsterdam, The Netherlands; 2 Department of Epidemiology and Biostatistics, Amsterdam Public Health Research Institute, Amsterdam UMC, VU University, Amsterdam, The Netherlands; 3 Department of Psychiatry, Amsterdam UMC, University of Amsterdam, Amsterdam, The Netherlands; 4 Translational Neurogenomics Group, QIMR Berghofer, Brisbane, Australia; Universidad Miguel Hernandez de Elche, SPAIN

## Abstract

**Aim:**

To investigate whether items of the SF-12, widely used to assess health outcome in clinical practice and public health research, provide unbiased measurements of underlying constructs in different demographic groups regarding gender, age, educational level and ethnicity.

**Methods:**

We included 23,146 men and women aged 18–70 of Dutch, South-Asian Surinamese, African Surinamese, Ghanaian, Turkish, or Moroccan origin from the HELIUS study. Both multiple group confirmatory factor analyses (MGCFA), with increasingly stringent model constraints (i.e. assessing Configural, Metric, Strong and Strict measurement invariance (MI)), and regression analysis were conducted to establish comparability of SF-12 items across demographic groups.

**Results:**

MI regarding gender, age and education was tested in the ethnic Dutch group (N = 4,615). In each subsequent step of testing MI, change in goodness-of-fit measures did not exceed 0.010 (RMSEA) or 0.004 (CFI). Moreover, goodness-of-fit indices showed good fit for strict invariance models: RMSEA<0.055; CFI>0.97. Regarding ethnicity, RMSEA values of metric and subsequent models fell above 0.055, indicating violation of measurement invariance in factor loadings, thresholds and residual variances. Regression analysis revealed possible age-, education- and ethnicity-related DIF. Adjustment for this DIF had little impact on the magnitude of age and educational differences in physical and mental health, but ethnic inequalities in physical health–and to a lesser extent mental health—were reduced after DIF adjustment.

**Conclusions:**

We found no evidence of violation of measurement invariance of the SF-12 regarding gender, age and educational level. If minor DIF would remain undetected in our MGCFA analyses, we showed that this would have negligible effect on the magnitude of demographic health inequalities. Regarding ethnicity, the SF-12 was not measurement invariant. After accounting for DIF, we observed a reduction of ethnic inequalities in health, in particular in physical health. Caution is warranted when comparing SF-12 scores across population groups with various ethnic backgrounds.

## Introduction

Health-related quality of life (HRQOL) has become an important outcome in public health monitoring and for the evaluation of treatment efficacy in clinical interventions [1]. HRQOL questionnaires are often used to compare physical or mental health status across groups based on certain demographic characteristics, such as age or educational level. However, it has been recognized that when people from different demographic groups think of, or value, certain aspects of health differently, this may influence their ratings and any comparison between these groups may be biased [2].

The SF-12 is an example of an HRQOL questionnaire, and is widely used for the assessment of general health [3]. It was developed in the US in the 1990s as a subset of the original SF-36 [4], since there was a need for a relatively brief though comprehensive health measure that can be used in large surveys of general as well as in specific population samples. The twelve items were selected from all eight subscales of the SF-36, i.e. general health perceptions, physical functioning, role limitations due to physical health problems, role limitations due to emotional health problems, bodily pain, vitality, general mental health, and social functioning [5]. The eight subscales can be comprised into two dimensions of health, known as the physical component summary (PCS) and the mental component summary (MCS). The final set of 12 questions was selected using data from a US population-based sample, and were validated in a US patient sample [5] and in US and European nationally representative population-based samples [6], based on their predictive value for these physical and mental summary scales.

Items of the SF-12 have been translated into numerous languages [6, 7], and physical and mental summary scores on the SF-12 were found to be reliable and valid in different settings. The SF-12 was shown to have satisfactory test-retest reliability and construct validity, i.e. the physical and mental summary scores obtained with the SF-12 closely resembled those obtained with the original SF-36 [5, 6]. However, the adequate validity and reliability do not necessarily imply that items of the SF-12 measure health aspects in a similar way across demographic groups. Cultural beliefs or expectations about health may lead to differences in the interpretation of specific questionnaire items or in different expectations about health. For example, when asked to rate their health in general, respondents may compare themselves with same-aged peers [8]. However, respondents with a lower educational level are likely to have same-aged friends or relatives who have relatively poor health, compared to respondents with a higher educational level. This might result in systematically better scores on reported health, given a similar health status, in those with lower levels of education. As a result, the difference in health status between those with a higher and lower educational level, which is well-established [9, 10], may be underestimated when using the SF-12 because of different frames of reference. To be able to make valid comparisons between groups with different individual characteristics, it must be investigated whether the measurement model of a health measurement instrument is equal in all groups, i.e. that the instrument is measurement invariant.

Measurement invariance is thus a desirable property of a measurement instrument, and implies that individuals’ characteristics that are unrelated to the construct of interest as measured by the scale do not affect individual item scores [2, 11]. In the case that measurement invariance does not hold, this implies that items function differentially (also known as DIF, differential item functioning) between groups. For example, subjects from different ethnic groups who have the same level of underlying physical functioning, should have the same probability of endorsing a specific answer category (e.g., ‘not limited’) if being asked whether they are limited in climbing stairs. DIF would imply that response probabilities are different, despite equal levels of physical functioning. Causes of DIF include different interpretation of items and differences in response style. When items exhibit DIF, ratings of these specific items may not be comparable and any comparison between subgroups would be biased if effect sizes of DIF are sufficiently large to be clinically relevant. It is therefore important to establish measurement invariance; this requires a systematic analysis of the correlational patterns across items.

In this study, we assess the measurement invariance of the SF-12 with respect to multiple demographic characteristics that are highly relevant in health-related research: gender, age, educational level, and ethnicity. Previous studies have used such an approach to investigate measurement invariance of the SF-12 or the SF-36 [12–18] and some of them indeed indicated a violation of the assumption of measurement invariance, with regard to one or more demographic characteristics [12–16]. Most of these studies focused on the SF-36 or were performed among specific patient groups [12–14, 16–18]. Fleishman and Lawrence [15] conducted the only study that explored DIF of the SF-12 in a general population sample. In this study, performed in the US, a comparison was made between white Americans, African-Americans and Hispanic Americans. Fleishman and Lawrence found age, gender, educational level, and ethnicity-related DIF. For example, with regard to the item measuring *feeling calm and peaceful*, participants with lower compared to higher educational level obtained higher scores on this item than would be expected based on their underlying mental health status. African and Hispanic American participants also gave higher ratings on this item, compared with white Americans with a similar health status. It was found that adjusting for DIF partly influenced the pattern of demographic differences in SF-12 scores. The results for these groups are not necessarily generalizable to European populations, however, no such studies have been performed in a European setting.

Given the wide-spread use of the SF-12, there is a need for further studies on its measurement invariance in the general population. Not only do European countries host different ethnic minority groups compared to the US, also educational or other subgroups within the majority population might differ, e.g. with respect to cultural beliefs. Therefore, the aim of this study is to examine measurement invariance of the SF-12 regarding age, gender, educational level, and ethnicity, using a multi-ethnic sample (HELIUS) of over 23,000 participants collected in the Netherlands.

## Methods

### Sample

The aim and design of the HELIUS (HEalthy LIfe in an Urban Setting) study have been described in detail elsewhere [19, 20]. In brief, the HELIUS study is a multi-ethnic cohort study conducted in Amsterdam, the Netherlands. Subjects were randomly, stratified by ethnicity, selected from the Amsterdam municipality register, and were sent an invitation letter (and a reminder after 2 weeks) by mail. We were able to contact 55% of those invited (55% among Dutch, 62% among Surinamese, 57% among Ghanaians, 46% among Turks, 48% among Moroccans), either by response card or after a home visit by an ethnically-matched interviewer. Of those, 50% agreed to participate (60% among Dutch, 51% among Surinamese, 61% among Ghanaians, 41% among Turks, 43% among Moroccans). Therefore, the overall response rate was 28% with some variations across ethnic groups. After a positive response, participants received a confirmation letter of the appointment for the physical examination, including a digital or paper version of the questionnaire (depending on the preference of the subject). In order to promote participation, non-Dutch people who did not respond to the written invitation letter were visited at home by an ethnically-matched interviewer. These interviewers provided additional information if needed (e.g. due to language or reading problems) or assisted in filling out the questionnaire in case the subject was willing to participate in the study.

Of 23,942 respondents who filled in the HELIUS questionnaire, we excluded respondents who did not belong to the six largest ethnic groups (n = 586). In addition, we excluded respondents with missing educational level (n = 208). Furthermore, we excluded two respondents who did not fill in any of the SF-12 items, while respondents who filled in at least one of the items were retained in the analyses (N = 914). The majority of them only missed one item (n = 700). The rate of missing at least one item varied between 1.8% in the Dutch origin group to 6.9% in the Ghanaian group.

The final sample consisted of 23,146 respondents of Dutch (n = 4,615), South-Asian Surinamese (N = 3,349), African Surinamese (N = 4,422), Ghanaian (N = 2,441), Turkish (N = 4,027) and Moroccan (N = 4,292) origin. Measurement invariance regarding age, gender and educational level was tested in the Dutch origin sample only (N = 4,615), as these variables, in particular education, were distributed unevenly across ethnic groups. Since controlling for covariates in MGCFA is not possible, analyzing measurement invariance regarding e.g. education level in the total population could have led to wrong conclusions about DIF regarding educational level, as this might actually have been caused by ethnicity. Measurement invariance regarding ethnicity was investigated in the total sample (N = 23,146). The Medical Ethics Committee of the Amsterdam Academic Medical Center (AMC) approved the study protocols. Written informed consent was obtained from all participants involved in the study.

### Measurements

We compared men and women, and five age groups: 18–30 years, 31–40 years, 41–50 years, 51–60 years and 61–70 years. The thresholds for these age categories were selected in such a way that all groups included roughly similar numbers of respondents. Participants were categorized into three educational levels, attained either in the Netherlands or in the country of origin: low education (never been to school, elementary schooling, lower vocational schooling, lower secondary schooling), intermediate education (intermediate vocational schooling or intermediate/higher secondary schooling (general)) or high education (higher vocational schooling or university).

Ethnicity was defined according to the country of birth of the participants as well as that of their parents [21]. Specifically, a participant was considered of non-Dutch ethnicity if either of the following criteria was fulfilled: (1) born outside the Netherlands and at least one parent born outside the Netherlands (i.e., first generation); or (2) born in the Netherlands, but both parents born outside the Netherlands (i.e., second generation). In addition, self-reported ethnicity was used to determine Surinamese subgroups (either African or South-Asian origin).

The version of the SF-12 employed with a 4-week time frame was included in the HELIUS questionnaire [6]. There were three different languages in which the SF-12 was administered: Dutch, English (for Ghanaians) and Turkish. The Dutch translation stems from the IQOLA project [7, 22], and was forward-backward translated into Turkish. Overall, there were three different modes of questionnaire completion: digital (43%) or paper version (31%), or paper version with interviewer assistance (26%). Participants in the Dutch and in both Surinamese groups completed the questions in Dutch. Of the Ghanaian and Turkish subsamples for which questionnaire language was ascertained, 78 and 33%, respectively, completed the questions in English or Turkish. Of the Moroccan subsample, about 33% were assisted by an interviewer who filled in the questionnaire in Dutch, but who often spoke Moroccan Arabic or Berber with the respondent. Unfortunately, no detailed information was available on the language of the interview of Moroccans. Sensitivity analyses were performed to examine if the SF-12 was measurement invariant regarding language (English vs. Dutch in Ghanaians (N = 2033) and Turkish vs. Dutch in Turks (N = 2766)) and interview mode (interview, paper or internet, N = 23146).

Four items that were reverse coded (items 1, 8, 9 and 10) were recoded to ensure that higher scores represent better health. For descriptive purposes, Physical and Mental Component Summary Scores (PCS and MCS) were calculated using previously published scoring coefficients [3]. Higher PCS and MCS indicate better health. For measurement invariance analyses, subsequent response categories of the SF-12 items were collapsed so that each response category contained ≥5% of the sample. For the Dutch sample, this resulted in 6 dichotomous items (items 2,3,4,5,6 and 7), one item with three categories (item 8) and five items with four categories (items 1, 9,10,11 and 12). For the total sample, this resulted in four dichotomous items (items 4,5,6 and 7), two items with three categories (items 2 and 3), two items with four categories (items 1 and 8) and four items with five categories (items 9,10,11 and 12) (S1 and S2 Tables). For the analyses on ethnicity, the Dutch sample had a similar number of response categories as the other ethnic groups.

### Measurement invariance analyses

In this study we applied multiple group confirmatory factor analysis (MGCFA) to investigate measurement invariance, because it enables the assessment of measurement invariance at different hierarchic levels, and in multiple groups at the same time.

Similar to confirmatory factor analysis (CFA), MGCFA requires a prespecified measurement model to be tested. The SF-12 was designed to reflect the physical and mental health dimensions, and numerous studies found that a two-factor model provided adequate fit to the data. We selected three two-factor models from the literature and compared their fit to the data of the Dutch participants. The best of these three models was used as the baseline model for subsequent measurement invariance tests. Model 1 specified items 1,2,3,4,5 and 8 to load on the physical factor, and items 6,7,9,10,11 and 12 on the mental factor [3]. The two factors were allowed to correlate, since it was shown that this generally provides better model fit [23, 24]. Model 2 additionally allowed cross-loadings for the items 1, 10 and 12, based on Fleishman and Lawrence’s findings [15]. In Model 3 we added residual covariances between all items derived from the same original SF-36 subscale: items 2 and 3 (physical functioning), items 4 and 5 (role physical), items 6 and 7 (role emotional) and items 9 and 11 (mental health) [24, 25]. Baseline models were identified by constraining the factor variances at 1 and the factor means at zero.

With MGCFA, the fit of models that have more constraints are compared with the fit of less constrained models. In other words, parameters that would be allowed to differ across groups in the less constrained model (Model 1), would be forced to be equal in the more constrained model (Model 2). In the case that Model 2 shows good fit and does not fit worse—based on model fit parameters—in comparison with Model 1, this indicates measurement invariance at the tested level (in this case Model 2). The fit of models can only be compared when the models are nested; i.e., when Model 1 can be transformed into Model 2 by imposing constraints on the parameters of Model 1. For each of the demographic variables (i.e., age, gender, educational level, and ethnicity), four hierarchic levels of measurement invariance were tested [11], which were described in detail elsewhere [26]. In short, analyses included testing of the following levels of invariance: 1) configural invariance, reflecting that the clustering of items and the factors that they represent is not different across groups, 2) metric invariance, indicating that factor loadings are comparable across groups, 3) strong invariance, reflecting that thresholds are comparable across groups, and 4) strict invariance, indicating that the residual variances are not significantly different across groups.

For all CFA analyses, we applied Weighted Least Squares Means and Variance adjusted (WLSMV) estimation with theta parameterization in Mplus version 7.4 [27] for statistical analysis with latent variables, in which the items were treated as ordinal variables [28]. For each successive step of MI testing, we applied the parameterization described in the Mplus manual [27].

Goodness-of-fit statistics were estimated for each model and for each model relative to the previous, less restricted, model. The chi-squared (χ^2^) statistic indicates the discrepancy between the covariance matrix of the observed data and the covariance matrix that is predicted by the factor model. When sample sizes are large, as in our study, a small difference between the two covariance matrices may already result in a significant value of χ^2^, even if the magnitude of this difference would not have practical or clinical implications [29, 30]. Therefore, and because it is recommended to use several indices simultaneously [31], we decided to also evaluate fit indices that favor more parsimonious models, such as the RMSEA (Root Mean Square Error of Approximation) and CFI (Comparative Fit Index) [32]. A better model fit is indicated by a low RMSEA value and a high CFI value. RMSEA values lower than 0.055 indicate good model fit [33]. CFI values higher than 0.97 indicate good model fit [34].

Regarding the successive steps in measurement invariance testing, we acknowledge that there is always some level of dissimilarity between groups, i.e. we do not expect that factor loadings and thresholds are identical between demographic groups. However, within measurement invariance research, certain criteria are used to indicate whether the level of dissimilarity is acceptable. Until recently, little was known about which criteria should be used when sample sizes are large, when more than two groups are compared at the same time, or when item responses are ordinal instead of continuous [29, 30, 35]. One recent study, however, focused on these particular situations and for the first time provided recommendations on the use of delta (Δ) goodness-of-fit measures (i.e. comparing the fit between more and less constrained models) for measurement invariance testing [33]. Following the recommendations of Rutkowski & Svetina [33], we considered declines in CFI larger than 0.004 and increases in RMSEA larger than 0.05 (metric invariance) or 0.01 (strong invariance) to indicate a significant worsening of fit. Testing of invariance of residual variances (strict models) was not performed by Rutkowski & Svetina, therefore we applied similar criteria as for the strong invariance models.

### Impact of DIF on demographic health inequalities

With MGCFA we tested whether differences in the factor structure (i.e., either at the level of factor loading, item thresholds, or residual item variances) were present. When testing for each subsequent level of measurement invariance, tests were performed simultaneously for all items. This method may therefore be less powerful to detect DIF of individual items [36]. Item-level DIF that may have been significant at a 1-degree of freedom test may go undetected when simultaneously tested with items that do not show DIF in a multiple-degree of freedom test. We therefore performed additional tests which were targeted at individual items to explore more subtle levels of DIF which may remain undetected by the MGCFA approach. In the case that significant DIF at the item level was found, we examined the impact that adjustment for this DIF had on the magnitude of physical and mental health inequalities across demographic groups. We expect to find inequalities in physical and mental health, for example to the disadvantage of ethnic minority groups, based on previous studies that showed large differences in disease prevalence [37–39]. In this additional analysis, we examined the relevance of adjusting for DIF when investigating physical and mental health differences between demographic groups. Two additional steps were performed for each demographic variable, which will be explained in the next two paragraphs, and which were described previously [26].

First, we conducted regression analysis to detect significant DIF at the item level. Using logistic regression analyses, we predicted each dichotomized item score with the corresponding factor score(s) from the strict invariance model and saved the residuals. Subsequently, we performed linear regression with the residuals from the logistic regression as the dependent variable, and ethnicity and ethnicity*factor score(s) as independent variables. This was done to conduct one overall test for uniform DIF (analogous to strong MI) and non-uniform DIF (analogous to metric MI), respectively [40]. Items with an explained variance (R^2^) of 2% or higher and significant uniform or non-uniform DIF (p-value below 0.05) were selected as items with DIF [40].

Second, to estimate the impact of adjusting for this DIF we returned to the MGCFA analysis. Adjustment for DIF was done by adapting strict invariance models (per demographic variable) so that for items with DIF, all threshold and, if necessary, factor loading constraints across groups were set free (‘partial strict’ models). Using means and variances of unadjusted and adjusted factor scores, from strict and partial strict models, respectively, we estimated two sets of standardized mean differences (Cohen’s d) across demographic groups. We evaluated whether 95% confidence intervals around d’s unadjusted for DIF and adjusted for DIF showed overlap, which would indicate that the significant DIF that was found had little impact on the magnitude of demographic health inequalities. Cohen’s d was calculated as (mean_1_—mean_2_) / √((sd(mean_1_)^2^ + sd (mean_2_)^2^)/2), and can be interpreted as small (d = 0.2), medium (d = 0.5) and large (d = 0.8) [41].

## Results

### Sample characteristics

Table 1 shows characteristics of the sample regarding gender, age, educational level and ethnicity. Higher PCS (P<0.01) and MCS (P<0.001) was found in men vs. women, in higher educated vs. lower educated groups (P<0.001), and in the Dutch vs. the other ethnic groups (P<0.001). Higher age was associated with higher MCS and lower PCS (P<0.001).

**Table 1:** Sample characteristics.

	N (%)	PCS (sd)^a^	MCS (sd)^a^
**Dutch origin sample (N = 4615)**			
Male gender	2119 (45.9%)	51.2 (7.2)	51.9 (8.2)
Female gender	2496 (54.1%)	50.6 (8.0)	50.2 (8.8)
18–30 years	873 (18.9%)	53.1 (5.5)	49.4 (8.4)
31–40 years	821 (17.8%)	52.6 (6.0)	50.1 (8.2)
41–50 years	943 (20.4%)	50.8 (7.9)	50.6 (9.0)
51–60 years	1098 (23.8%)	49.6 (8.3)	51.2 (9.0)
61–70 years	880 (19.1%)	48.7 (8.7)	53.3 (7.6)
High education	2784 (60.3%)	52.1 (6.7)	51.2 (8.0)
Medium education	1018 (22.1%)	50.6 (7.6)	50.3 (9.0)
Low education	813 (17.6%)	47.1 (9.4)	50.9 (9.7)
**Total sample (N = 23146)**			
Dutch	4615 (19.9%)	50.9 (7.6)	51.0 (8.6)
South-Asian Surinamese	3349 (14.5%)	46.5 (9.8)	47.4 (10.9)
African Surinamese	4422 (19.1%)	48.3 (9.0)	50.0 (10.0)
Ghanaian	2441 (10.5%)	47.9 (8.8)	49.3 (9.5)
Turkish	4027 (17.4%)	45.5 (10.6)	44.9 (11.1)
Moroccan	4292 (18.5%)	46.2 (10.2)	45.9 (10.7)

^a^Standard PCS and MCS were computed for people with complete data on the SF-12 or with 1 item missing. Respondents with 2 or more missings were excluded (N = 193), but not in subsequent analyses.

### Measurement invariance analyses

**Fig 1:**
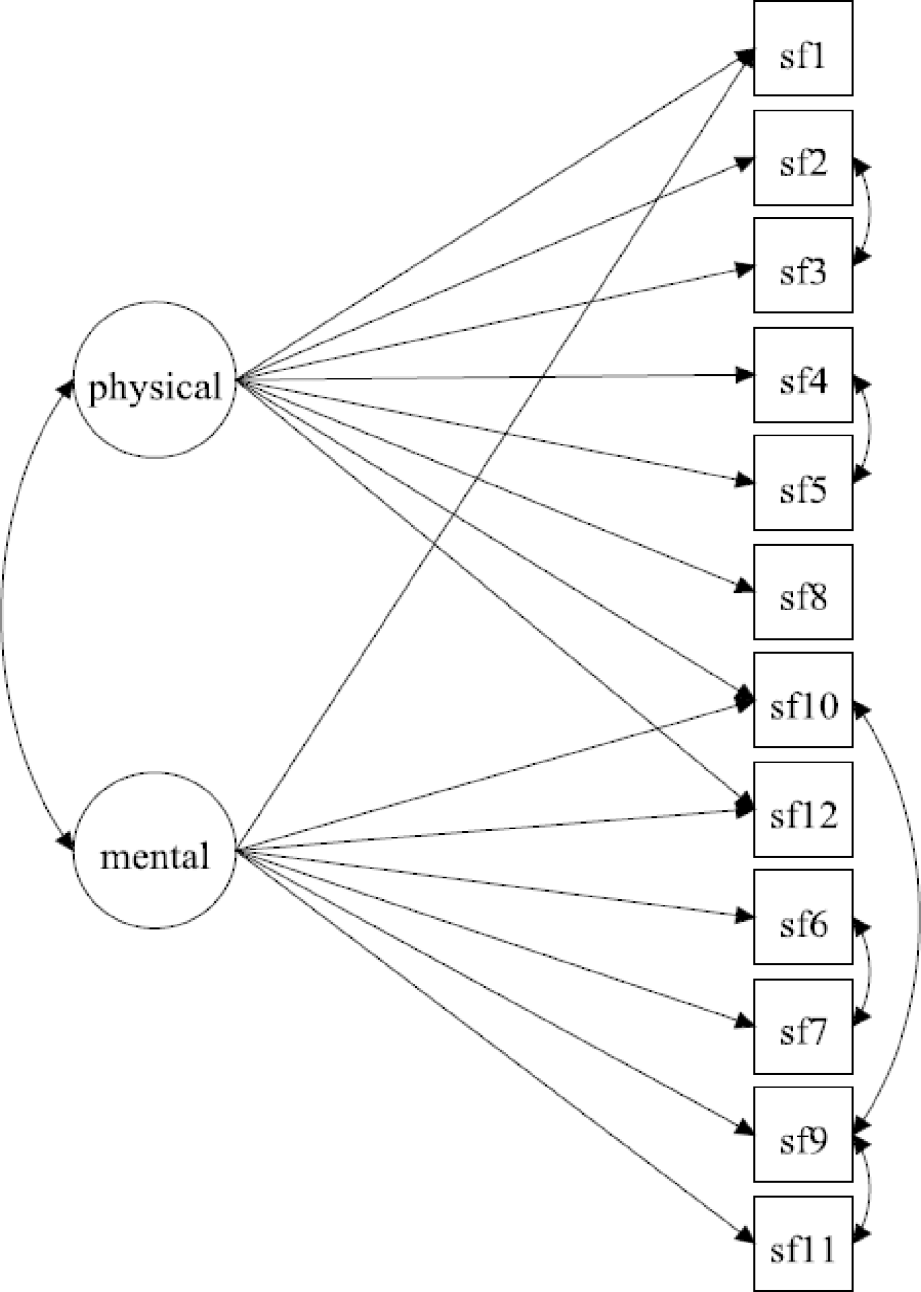
Baseline factor model of the SF-12 that was used to assess measurement invariance across demographic groups.

In Table 2, goodness-of-fit indices are compared between potential baseline models (Models 1–3). The best fitted model was model 3, both in the Dutch sample and in the total sample: **χ**^**2**^ and RMSEA values were lower and CFI values were higher, when compared to Model 1 and 2. Modification indices suggested that this model could be improved by adding a fifth residual correlation between items 9 (calm and peaceful) and 10 (downhearted and blue). Model 4 added this correlation, and since this model showed good fit (RMSEA: 0.047 and 0.046, CFI: 0.991 and 0.995 in the Dutch and total sample, respectively), no further improvements were made. This model mostly showed good fit in the study population stratified by age, gender, educational level, and ethnicity (Table 3). Only in Ghanaian, Turkish and Moroccan subgroups RMSEA values were above the threshold of 0.055, but since model fit in the total population was good, we decided to use model 4 as our baseline model in subsequent analyses of measurement invariance (Fig 1).

**Table 2:** Comparison of model fit for baseline model.

	Number of free parameters	χ^2^ (df)	RMSEA	CFI
**Dutch sample**				
Model 1^a^	Total: 37 Factor loadings: 12 Thresholds: 24 Factor covariance: 1	2791.368 (53)*	0.106 (0.102–0.109)^b^	0.946^b^
Model 2	Total: 40 Model 1 + 3 factor loadings	1189.533 (50)*	0.070 (0.067–0.074)^b^	0.978
Model 3	Total: 44 Model 2 + 4 item covariances	677.946 (46)*	0.055 (0.051–0.058)	0.988
Model 4 (Model for MI testing)	Total: 45 Model 3 + 1 item covariance	507.833 (45)*	0.047 (0.044–0.051)	0.991
**Total sample**				
Model 1^a^	Total: 43 Factor loadings: 12 Thresholds: 30 Factor covariance: 1	15008.851 (53)*	0.110 (0.109–0.112)^b^	0.967^b^
Model 2	Total: 46 Model 1 + 3 factor loadings	12646.343 (50)*	0.104 (0.103–0.106)^b^	0.972
Model 3	Total: 50 Model 2 + 4 item covariances	5284.281 (46)*	0.070 (0.069–0.072)^b^	0.988
Model 4 (Model for MI testing)	Total: 51 Model 3 + 1 item covariance	2264.403 (45)*	0.046 (0.045–0.048)	0.995

* P < .001

^a^ Model 1:6 items load on the physical factor and 6 on the mental factor; factors are allowed to co-vary. The model is identified by constraining factor variances and means at 1 and 0, respectively. Model 2: 3 items (1, 10 and 12) load on both factors; Model 3: residuals of 4 item pairs belonging to the same subscale (i.e. 2&3, 4&5, 6&7, 9&11) are allowed to co-vary; Model 4: residuals of item pair 9 & 10 are allowed to co-vary

^b^ Poor model fit (RMSEA>0.055; or CFI<0.97)

**Table 3:** Model fit of the baseline model in each of the demographic groups.

	Free parameters^a^	χ^2^ (df)	RMSEA	CFI
**Dutch origin sample**				
Male gender	45	266.534 (45)*	0.048 (0.043–0.054)	0.989
Female gender	45	308.392 (45)*	0.048 (0.043–0.054)	0.991
18–30 years	45	108.338 (45)*	0.040 (0.031–0.050)	0.986
31–40 years	45	113.968 (45)*	0.043 (0.033–0.053)	0.990
41–50 years	45	132.941 (45)*	0.046 (0.037–0.055)	0.994
51–60 years	45	184.965 (45)*	0.053 (0.045–0.061)	0.991
61–70 years	45	131.199 (45)*	0.047 (0.037–0.056)	0.995
High education	45	291.627 (45)*	0.044 (0.040–0.049)	0.989
Medium education	45	143.597 (45)*	0.046 (0.038–0.055)	0.991
Low education	45	135.339 (45)*	0.050 (0.040–0.059)	0.995
**Total sample**				
Dutch	51	501.341 (45)*	0.047 (0.043–0.051)	0.991
South-Asian Surinamese	51	347.623 (45)*	0.045 (0.040–0.049)	0.996
African Surinamese	51	449.622 (45)*	0.045 (0.041–0.049)	0.996
Ghanaian	51	403.428 (45)*	0.057 (0.052–0.062)^b^	0.990
Turkish	51	748.754 (45)*	0.062 (0.058–0.066)^b^	0.992
Moroccan	51	687.716 (45)*	0.058 (0.054–0.062)^b^	0.993

* P < .001

^a^ Unequal number of parameters because in total sample more thresholds were estimated. The model is identified by constraining factor variances and means at 1 and 0, respectively

^b^ Poor model fit (RMSEA>0.055; or CFI<0.097)

Results from MGCFA regarding gender, age and educational level are shown in Table 4. Configural models showed good fit for all three variables. In addition, adding constraints for equal factor loadings (metric invariance), item thresholds (strong invariance) and residual variances (strict invariance) did not lead to reduced model fit. The final strict invariance model showed good fit, while ΔRMSEA and ΔCFI for increasingly stringent test of measurement invariance never exceeded the critical values. With regard to ethnicity (Table 4), the configural invariance model had–according to its RMSEA value—good model fit (i.e. RMSEA was 0.052), but the strong and strict invariance models did not show adequate fit. This indicates that not all factor loadings, thresholds and residual variances are measurement invariant across ethnic groups. Delta goodness-of-fit indices resembled this: the change from metric to strong and strong to strict invariance models resulted in declines in CFI that were larger than the critical value of 0.004.

**Table 4:** Measurement invariance tests regarding age, gender and educational level (Dutch origin sample, N = 4,615) and ethnicity (Total sample, N = 23,146).

	Model	Free parameters	χ^2 ^(df)	RMSEA	CFI
**Gender**	1.Configural	90	573.770 (90)*	0.048 (0.045–0.052)	0.991
	2.Metric	77	563.143 (103)*	0.044 (0.040–0.048)	0.991
	3.Strong	67	588.737 (113)*	0.043 (0.039–0.046)	0.991
	4.Strict	50	584.855 (130)*	0.039 (0.036–0.042)	0.991
**Age**	1.Configural	225	662.373 (225)*	0.046 (0.042–0.050)	0.993
	2.Metric	173	783.001 (277)*	0.044 (0.041–0.048)	0.992
	3.Strong	133	1034.643 (317)*	0.050 (0.046–0.053)	0.988
	4.Strict	65	1105.714 (385)*	0.045 (0.042–0.048)	0.988
	5.Partial strict: thresholds items 3,4,5 free^c^	77	931.761 (373)*	0.040 (0.037–0.044)	0.991
**Education**	1.Configural	135	585.030 (135)*	0.047 (0.043–0.050)	0.991
	2.Metric	109	628.522 (161)*	0.043 (0.040–0.047)	0.991
	3.Strong	89	801.276 (181)*	0.047 (0.044–0.051)	0.988
	4.Strict	55	801.142 (215)*	0.042 (0.039–0.045)	0.989
	5.Partial strict: thresholds items 4,5 free^c^	59	705.657 (211)*	0.039 (0.036–0.042)	0.990
**Ethnicity**	1.Configural	306	3099.841 (270)*	0.052 (0.050–0.054)	0.994
	2.Metric	241	4912.339 (335)*	0.060 (0.058–0.061)^a^	0.990
	3.Strong	161	6915.230 (415)*	0.064 (0.062–0.065)^a^	0.986^b^
	4.Strict	76	10421.327 (500)*	0.072 (0.071–0.073)^a^	0.978^b^
	5.Partial strict: thresholds and loadings items 9,3,5,2,1,6,10 free^c^	216	4250.544 (345)*	0.054 (0.053–0.056)	0.992

* P<0.001

^a^ Poor model fit (RMSEA>0.055; or CFI<0.97)

^b^ Significant worsening of fit compared to previous model (increase in RMSEA>0.05 (metric) or >0.01 (strong/strict); or decline in CFI>0.004)

^c^ Based on logistic regression results, we relaxed the constraints on thresholds—and factor loadings if necessary—of items that showed DIF until the partial strict model showed good fit.

Sensitivity analyses were performed with regard to interview mode in the total sample (configural, metric and strong models showed good fit, but strict model showed poor fit), regarding Turkish vs. Dutch language in Turkish participants (configural model had good fit, but metric, strong and strict models had poor fit) and regarding English vs. Dutch language in Ghanaian participants (configural to strict models showed poor fit). These analyses indicated that the SF-12 was largely measurement invariant for interview mode, but not for language of questionnaire completion within Turkish and Ghanaian participants (S3 and S4 Tables).

### Impact of DIF on demographic health inequalities

The additional regression analyses, targeted at individual items, revealed no items with gender-related DIF, three items with age-related DIF (items 3, 4 and 5), two items with education-related DIF (items 4 and 5), and one item with ethnicity-related DIF (item 9). We examined the impact that this DIF had on the final strict measurement invariance models, by adjusting these models in such a way that the thresholds and, if necessary, factor loadings of items with DIF were set free across groups. Table 4 shows good model fit (based on RMSEA and CFI) for the partial strict models. For age and education the strict model already showed good fit, and therefore we only relaxed the threshold constraints on items that were indicated by the regression analysis for that specific grouping variable. However, for ethnicity, it was necessary to relax more constraints on thresholds and factor loadings. Only when loadings and thresholds for items 9,3,5,2,1, 6 and 10 were relaxed (in order of the highest explained variance in regression models) the partial strict model for ethnicity showed good fit. Results (S7 and S8 Tables) indicate that factor loadings of items 1,2,3 and 9 differed between Ghanaians and the other groups in particular. Item 10 appeared more important for the physical factor than for the mental factor in Turkish, Moroccan and Ghanaian groups, whereas it was the other way around in the Dutch and both Surinamese groups. With regard to item thresholds, items 1,9 and 10 were scored more often with extreme low and high responses in ethnic minority groups compared to the Dutch. Items 2,3 and 6 were scored lower in Ghanaians and higher in Dutch, given similar underlying physical and mental health.

**Fig 2:**
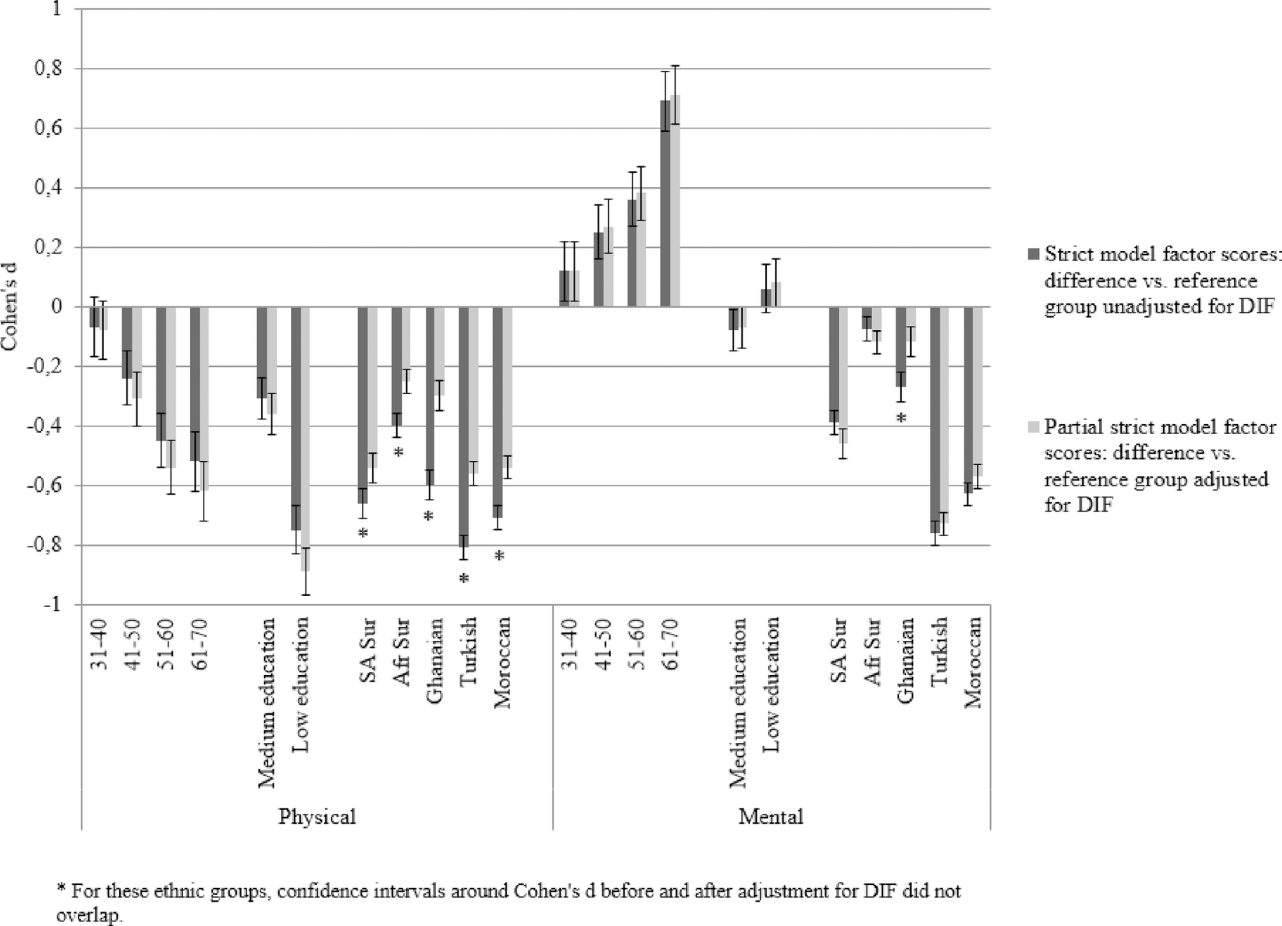
Standardized mean differences (Cohen’s d) in physical and mental health, before and after adjustment for DIF. Reference groups are 18-30-year-olds, high educated and Dutch participants, respectively.

To evaluate the relevance of this misfit–in particular for ethnicity—Fig 2 shows standardized differences (Cohen’s d) in physical and mental health between demographic groups, based on factor scores obtained in the strict models, that did not adjust for DIF, and the partial strict models, that did adjust for DIF. Mean differences in physical and mental health between age, education and ethnic groups are substantial, as expected. However, confidence intervals of the effect sizes did overlap between DIF adjusted and DIF unadjusted models for age and education. This indicates that the magnitude of mental and physical health differences across age groups and educational levels does not change substantially when DIF is taken into account. For ethnicity, we observed significant changes in effect size, indicated by confidence intervals that did not overlap: ethnic differences in physical health between ethnic minority groups and the Dutch group were reduced after DIF was taken into account. For mental health, this was only the case for the comparison between Ghanaian and Dutch participants.

## Discussion

Empirical evidence of measurement invariance is essential for making valid health comparisons across demographic groups. In this article, we examined whether the measurement model of the SF-12 is equal across demographic groups, in a large general population sample including over 23,000 participants. Our results based on MGCFA analysis indicated no violation of measurement invariance with respect to gender, age or educational level. If minor violation of measurement invariance would go undetected in our MGCFA analyses, we showed that this would have a negligible effect on the magnitude of demographic differences in physical and mental health scores. With regard to ethnic background, we did observe violation of measurement invariance, and after taking into account this DIF we observed smaller ethnic differences in physical and–to some extent–mental health.

Fleishman and Lawrence [15] conducted the only measurement invariance study so far on the SF-12 in a general population sample. They reported DIF with respect to age, gender, education and ethnicity, but only age- and ethnicity-related DIF changed conclusions regarding between-group differences in physical or mental health, either with regard to the significance or the direction of associations. Ethnicity-related DIF was found by Fleishman and Lawrence in items 1,5,7,9,11 and 12, of which the most prominent was item 9 ‘*feeling calm and peaceful’*, which also showed significant DIF in our analyses. Nevertheless, comparisons with their results should be made with caution, since quite different ethnic groups are being compared. Other studies were performed on the SF-36, or in specific population groups, and some of these also found evidence for DIF of several items that are included in the SF-12 [12–14, 16, 18].

Our study was the first to employ MGCFA to assess measurement invariance of the SF-12. MGCFA has the advantage over other applied methods that it assesses different levels of measurement invariance [42]. In addition, the criteria that we applied to evaluate the statistical significance of violation of measurement invariance (absolute values of RMSEA and changes in RMSEA and CFI) were shown to be less sensitive to sample size, as compared to fit indices that are directly based on χ^2^ [32]. Our assessment of model fit may have led to lower sensitivity to detect small DIF effects, as compared to previous studies. This effect was somewhat attenuated since we applied criteria that were more strict–and thus more sensitive to detect DIF–in comparison with commonly used criteria for MGCFA [33]. Still, additional logistic regression analyses indicated age- and education-related DIF that was not observed with MGCFA, which corrobrates findings from a simulation study [36]. The clinical relevance of this age- and education-related DIF–even though statistically significant—appeared to be low, given that the magnitude of between-group inequalities in physical and mental health did not change when taking DIF into account. Most of the studies cited above also showed that only part of the DIF effects were meaningful [12], or that statistically significant DIF did not translate into substantial differences in the pattern of SF-12 physical and mental health scores across demographic groups [13–15, 18]. For ethnicity-related DIF, however, this appeared different in our study: ethnic inequalities in physical health were substantially reduced after taking into account DIF in six items, and this applied to all comparisons between ethnic minority groups vs. the Dutch. For mental health only the difference between Ghanaian and Dutch participants was significantly reduced after DIF adjustment.

Although the validity and feasibility of the SF-12 has been examined in many studies, e.g. [6, 24, 43, 44], most researchers use the SF-12 as an outcome measure without examining whether the instrument performs similarly across the groups that are under study, e.g. [45–48]. Thus, in such studies an implicit assumption is made that health inequalities observed based on SF-12 measurements are valid representions of true underlying health inequalities. The results of our study provided indications for measurement invariance with regard to age, gender and education. Thus, we may conclude that the health inequalities we and others have observed when comparing different age groups, genders and those with different educational levels indeed reflect differences in underlying physical and mental health, and are not the result of DIF. The observed health inequalities mostly corroborate findings from previous studies, either based on subjective measures such as the SF-12, or on more objective health indicators, such as disease prevalence and mortality. They reflect the greater vulnerability to physical and mental health problems in specific demographic groups: women, those with a higher age (except that they had better mental health summary scores compared to younger adults) and those with lower education [14, 15, 49–52].

Measurement invariance of the SF-12 did not hold with regard to ethnicity. We observed that ethnic inequalities in physical health, and to some extent mental health, were reduced after DIF adjustment. Thus, the inequalities obtained when using the SF-12 should be interpreted with caution. In our study, factor loadings and thresholds for multiple items had to be relaxed between ethnic groups in order to obtain a good fitting model. This indicates that influences other than the latent physical and mental health factors also determine ethnic differences in item responses, in particular in the Ghanaian group. These influences might in part be health-related, for example when health is conceptualized differently, and in part not health-related, for example if the likelihood certain response option are chosen differs between groups. Strategies for dealing with DIF may include removing items that show DIF, but for short instruments such as the SF-12 this might lead to reduced coverage of important health domains as more than half of the items is affected by DIF. We would recommend to apply latent-variable analysis when investigating SF-12 scores in multi-ethnic samples, as this allows taking DIF into account. Further investigation is needed, for example, qualitative methods may be used to study whether ethnic minority groups indeed interpret or understand these items in such a way that they are scored worse compared to Dutch respondents. This also applies to participants who completed the SF-12 in English or in Turkish. Our additional analysis indicated that these groups cannot be directly compared with participants completing the questionnaire in Dutch.

The results of this study should be interpreted in view of the following limitations. First, all studies on measurement invariance of health instruments to some extent aim to differentiate between ‘inequalities in SF-12 scores attributable to true health’, and ‘inequalities in SF-12 not attributable to true health’, but we have to acknowledge that ‘true health’ is not measurable. This entails an inherent limitation in all research that aims to uncover between-group differences in reporting of health problems [53–55]. In view of this limitation, our conclusion–that the fit of the specified factor model for the SF-12 did not differ substantively between gender, age and educational groups–does not imply that there are no differences at all between these groups in the concept of health. Several aspects of health, for example the presence of specific diseases, or lifestyle factors, were not measured in this study. If we would have used an alternative instrument to operationalize health, the conclusion may have been different. Furthermore, health aspects that are relevant in one or more of these demographic groups, may not be represented by items of the SF-12. This would imply that content validity does not hold for all groups. Future studies, employing for example a qualitative approach, may evaluate this important aspect of validity further.

Second, our response rate was relatively low, possibly resulting in selection bias. E.g. non-responders to our study might be in particular individuals with the most differential view on health, or the lowest proficiency of the Dutch language. However, we were able to include large numbers of each ethnic group in which all social-economic levels are represented, and non-response analyses show that socio-economic differences between participants and non-participants were very small [20]. In addition, respondents were allowed to use that mode of response with which they were most comfortable; in particular, those individuals who received assistance from a trained interviewer of similar ethnic background would have misconceptions corrected. Such an approach is common in epidemiological research, to avoid disinterest and misunderstanding when using health questionnaires such as the SF-12. The option of assistance by an interviewer was offered with the aim to promote response rates in these groups, thereby reducing selection bias. Nonetheless, the approach might have taken away some of the language and culture-related variance that would have emerged when each participant had the same mode of interview. In our sensitivity analyses we indeed found that there is variance between Dutch-speaking and non-Dutch-speaking groups who completed the SF-12. Unfortunately, with regard to Moroccan respondents we had no detailed information on the language spoken during the interviews. To obtain further insight into the role of culture in how people respond to questions of the SF-12, future research may examine measurement invariance regarding different lengths of stay in the Netherlands, as immigrants with a longer length of stay may be more acculturated compared to those with a shorter length of stay. In addition, research should be conducted in the specific ethnic minority groups, to examine differences in the interpretation health in general, and the SF-12 in particular.

Third, for the calculation of standardized differences we made use of factor scores. WLSMV models ignore residual covariances in the computation of factor scores. Therefore, we compared our WLSMV factor scores with factor scores derived from a maximum likelihood model (taking into account residual covariances, but not treating the outcome variables as ordinal). Physical and mental factor scores from both estimation methods correlated >.97. We concluded that the influence of ignoring residual covariances for the calculation of factor scores is limited.

A fourth limitation of this study is that little is known about which criteria for measurement invariance should be used when sample sizes are large, when more than two groups are compared at the same time, or when item scores are ordinal instead of quantitative [29, 30, 35]. We applied criteria that were recently recommended, but these were developed based on simulation studies of unidimensional scales, and not of multidimensional scales such as the SF-12 [33]. More research can be done in the field of measurement invariance, to guide researchers regarding which methods and criteria for significant and relevant DIF should or should not be applied. For example, it was only recently suggested that CFI has a low ability to detect model misspecification [33], while most measurement invariance research has used CFI as an indicator of goodness-of-fit. Our study evaluated both RMSEA and CFI, and our results indeed indicated that only RMSEA pointed at model misspecification, while CFI did not.

Fifth, our method of evaluating DIF required a grouping variable. We found measurement invariance for age groups, but it might be that analyses in which age was treated as a continuous variable would have resulted in different conclusions.

To conclude, we found no evidence of violation of measurement invariance of the SF-12 regarding age, gender, and educational level in a sample of people from Dutch origin aged 18–70. If minor violation of DIF would remain undetected in our MGCFA analyses, we showed that this would have negligible effect on the magnitude of demographic inequalities in physical and mental health. However, the questionnaire was not measurement invariant regarding ethnicity in a multi-ethnic population, and adjustment for this DIF resulted in reduced ethnic inequalities in physical and–to a lesser extent–mental health. As such, our results confirm the appropriateness of the SF-12 to assess physical and mental health differences across age groups, gender and educational levels, but caution is warranted when interpreting differences between subpopulations with various ethnic backgrounds.

## Supporting information

S1 Table Item responses by gender, education and age (after merging categories with less than 5 responses in the Dutch sample). (DOCX)

S2 Table Item responses by ethnic group (after merging categories with <5% in the total sample). (DOCX)

S3 Table Sensitivity analyses: model fit in separate groups regarding interview mode and language. (DOCX)

S4 Table Sensitivity analyses: measurement invariance analyses regarding interview mode and language. (DOCX)

S5 Table Linear regression with residuals from logistic regression as the outcome and demographic variables as predictors. (DOCX)

S6 Table Linear regression with residuals from logistic regression as outcome and demographics and demographics*factor scores as predictors. (DOCX)

S7 Table Factor means and variances, and standardized mean differences, in strict and partial strict multiple group confirmatory factor models. (DOCX)

S8 Table Unstandardized results partial strict model for ethnicity. (DOCX)

S9 Table Standardized results partial strict model for ethnicity. (DOCX)
