# Supplementary material for: Measurement invariance of the SF-12 among different demographic groups: The HELIUS study
Source: PLoS One. 2018 Sep 13;13(9):e0203483. doi: 10.1371/journal.pone.0203483 (PMC6136718; doi:10.1371/journal.pone.0203483)
Supplement: S1 Table — (DOCX) [file pone.0203483.s001.docx]

**S1 Table. Item responses by gender, education and age (after merging categories with less than 5 responses in the Dutch sample)**

|  | **Item** | **Response category** | **Men**  **N=2119** | **Women**  **N=2496** | **Low education**  **N=813** | **Mid education**  **N=1018** | **High education**  **N=2784** | **18-30 y**  **N=873** | **31-40 y**  **N=821** | **41-50 y**  **N=943** | **51-60 y**  **N=1098** | **61-70 y**  **N=880** |
| --- | --- | --- | --- | --- | --- | --- | --- | --- | --- | --- | --- | --- |
| 1 | General Health | Poor / fair | 8.8% | 10.2% | 22.9% | 10.3% | 5.4% | 4.1% | 3.7% | 10.0% | 13.8% | 14.9% |
|  |  | Good | 46.9% | 49.4% | 57.9% | 52.8% | 43.7% | 39.6% | 44.6% | 47.9% | 50.8% | 57.2% |
|  |  | Very good | 30.9% | 29.8% | 14.7% | 27.7% | 35.8% | 40.8% | 38.1% | 29.4% | 25.5% | 19.7% |
|  |  | Excellent | 13.4% | 10.6% | 4.6% | 9.1% | 15.0% | 15.5% | 13.6% | 12.6% | 9.9% | 8.3% |
| 2 | Limited in moderate activities | Yes, limited a lot / a little | 9.7% | 14.4% | 27.6% | 13.1% | 7.5% | 5.0% | 4.9% | 11.0% | 16.1% | 23.0% |
|  |  | No, not limited at all | 90.3% | 85.6% | 72.4% | 86.9% | 92.5% | 95.0% | 95.1% | 89.0% | 83.9% | 77.0% |
| 3 | Limited in climbing several flights | Yes, limited a lot / a little | 13.0% | 16.7% | 33.0% | 18.0% | 8.7% | 6.3% | 4.4% | 13.0% | 19.6% | 29.9% |
|  |  | No, not limited at all | 87.0% | 83.3% | 67.0% | 82.0% | 91.3% | 93.7% | 95.6% | 87.0% | 80.4% | 70.1% |
| 4 | Accomplished less physical | Yes | 16.6% | 19.8% | 26.0% | 19.4% | 15.7% | 14.1% | 17.0% | 19.4% | 20.6% | 19.7% |
|  |  | No | 83.4% | 80.2% | 74.0% | 80.6% | 84.3% | 85.9% | 83.0% | 80.6% | 79.4% | 80.3% |
| 5 | Limited in work or daily activities | Yes | 17.7% | 20.1% | 28.6% | 20.2% | 15.8% | 12.8% | 15.0% | 20.1% | 23.6% | 22.0% |
|  |  | No | 82.3% | 79.9% | 71.4% | 79.8% | 84.2% | 87.2% | 85.0% | 79.9% | 76.4% | 78.0% |
| 6 | Accomplished less emotional | Yes | 10.8% | 15.0% | 16.2% | 15.3% | 11.4% | 14.5% | 12.8% | 14.6% | 14.0% | 9.3% |
|  |  | No | 89.2% | 85.0% | 83.8% | 84.7% | 88.6% | 85.5% | 87.2% | 85.4% | 86.0% | 90.7% |
| 7 | Not careful as usual | Yes | 9.7% | 12.0% | 13.0% | 12.7% | 9.8% | 11.7% | 11.7% | 11.3% | 13.0% | 6.7% |
|  |  | No | 90.3% | 88.0% | 87.0% | 87.3% | 90.2% | 88.3% | 88.3% | 88.7% | 87.0% | 93.3% |
| 8 | How much did pain interfere | Extremely / Quite a bit / Moderately | 7.4% | 9.5% | 18.7% | 9.2% | 5.4% | 4.4% | 5.0% | 9.9% | 11.7% | 10.7% |
|  |  | A little bit | 31.9% | 36.2% | 38.1% | 35.5% | 32.6% | 30.9% | 32.2% | 33.7% | 37.7% | 35.5% |
|  |  | Not at all | 60.7% | 54.3% | 43.2% | 55.4% | 62.0% | 64.7% | 62.8% | 56.4% | 50.6% | 53.8% |
| 9 | Felt calm and peaceful | None / A little / Some of the time | 12.7% | 19.7% | 19.8% | 19.3% | 14.5% | 20.8% | 18.9% | 19.2% | 14.4% | 9.6% |
|  |  | A good bit of the time | 24.5% | 29.0% | 21.9% | 26.4% | 28.6% | 29.9% | 31.9% | 27.6% | 25.0% | 21.1% |
|  |  | Most of the time | 51.4% | 44.8% | 46.4% | 45.9% | 49.0% | 45.5% | 44.0% | 44.7% | 51.8% | 52.2% |
|  |  | All of the time | 11.4% | 6.4% | 11.8% | 8.5% | 7.9% | 3.8% | 5.2% | 8.4% | 8.8% | 17.1% |
| 10 | Have a lot of energy | None / A little of the time | 4.5% | 5.6% | 8.8% | 6.1% | 3.7% | 4.0% | 4.5% | 6.6% | 5.6% | 4.4% |
|  |  | Some of the time | 20.7% | 25.5% | 29.7% | 25.0% | 20.8% | 24.1% | 23.0% | 23.7% | 23.4% | 22.2% |
|  |  | A good bit of the time | 33.5% | 33.0% | 26.1% | 31.1% | 36.1% | 37.4% | 40.1% | 32.0% | 30.8% | 27.1% |
|  |  | Most of the time | 34.2% | 30.9% | 27.5% | 31.9% | 34.1% | 31.5% | 29.8% | 32.0% | 32.8% | 35.9% |
|  |  | All of the time | 6.9% | 5.1% | 7.9% | 6.0% | 5.4% | 3.0% | 2.6% | 5.8% | 7.4% | 10.4% |
| 11 | Felt downhearted and blue | All / Most / A good bit of the time | 5.6% | 7.8% | 9.2% | 9.5% | 5.1% | 9.1% | 6.1% | 8.3% | 6.5% | 4.1% |
|  |  | Some of the time | 26.8% | 32.9% | 28.3% | 31.1% | 30.2% | 32.9% | 34.1% | 28.4% | 31.1% | 24.0% |
|  |  | A little of the time | 40.4% | 38.1% | 32.9% | 36.3% | 42.0% | 45.1% | 41.5% | 36.7% | 34.2% | 39.7% |
|  |  | None of the time | 27.2% | 21.2% | 29.6% | 23.0% | 22.7% | 13.0% | 18.3% | 26.6% | 28.1% | 32.3% |
| 12 | Health problems interfere with social activities | All / Most / A good bit of the time | 5.4% | 6.3% | 10.7% | 6.8% | 4.1% | 5.3% | 3.8% | 7.1% | 7.1% | 5.5% |
|  |  | Some of the time | 11.6% | 17.7% | 17.7% | 15.9% | 13.7% | 12.9% | 12.5% | 16.4% | 16.4% | 15.5% |
|  |  | A little of the time | 21.9% | 24.0% | 20.2% | 22.8% | 24.0% | 27.3% | 27.0% | 20.3% | 20.3% | 21.5% |
|  |  | None of the time | 61.1% | 52.0% | 51.4% | 54.5% | 58.3% | 54.5% | 56.6% | 56.2% | 56.1% | 57.6% |
