# Supplementary material for: Measurement invariance of the SF-12 among different demographic groups: The HELIUS study
Source: PLoS One. 2018 Sep 13;13(9):e0203483. doi: 10.1371/journal.pone.0203483 (PMC6136718; doi:10.1371/journal.pone.0203483)
Supplement: S2 Table — (DOCX) [file pone.0203483.s002.docx]

**Appendix Table 2. Item responses by ethnic group (after merging categories with <5% in the total sample)**

|  | **Item** | **Response category** | **Dutch**  **N=4615** | **South-Asian Surinamese**  **N=3349** | **African Surinamese**  **N=4422** | **Ghanaian**  **N=2441** | **Turkish**  **N=4027** | **Moroccan**  **N=4292** |
| --- | --- | --- | --- | --- | --- | --- | --- | --- |
| 1 | General Health | Poor / fair | 9.6% | 30.9% | 22.3% | 19.6% | 33.9% | 36.9% |
|  |  | Good | 48.2% | 52.8% | 56.3% | 50.7% | 51.3% | 48.8% |
|  |  | Very good | 30.3% | 10.6% | 13.8% | 19.5% | 11.0% | 9.9% |
|  |  | Excellent | 11.9% | 5.7% | 7.7% | 10.2% | 3.8% | 4.5% |
| 2 | Limited in moderate activities | Yes, limited a lot | 1.2% | 6.1% | 3.2% | 10.6% | 11.2% | 6.6% |
|  |  | Yes, limited a little | 11.1% | 27.4% | 21.0% | 26.7% | 30.1% | 27.5% |
|  |  | No, not limited at all | 87.7% | 66.5% | 75.8% | 62.7% | 58.7% | 65.9% |
| 3 | Limited in climbing several flights | Yes, limited a lot | 1.2% | 5.8% | 3.8% | 13.0% | 11.6% | 7.1% |
|  |  | Yes, limited a little | 13.8% | 28.2% | 26.4% | 28.9% | 31.0% | 27.7% |
|  |  | No, not limited at all | 85.0% | 66.0% | 69.9% | 58.2% | 57.4% | 65.3% |
| 4 | Accomplished less physical | Yes | 18.3% | 31.9% | 23.8% | 21.2% | 35.6% | 32.4% |
|  |  | No | 81.7% | 68.1% | 76.2% | 78.8% | 64.4% | 67.6% |
| 5 | Limited in work or daily activities | Yes | 19.0% | 30.6% | 23.0% | 19.8% | 33.9% | 30.7% |
|  |  | No | 81.0% | 69.4% | 77.0% | 80.2% | 66.1% | 69.3% |
| 6 | Accomplished less emotional | Yes | 13.1% | 26.4% | 17.8% | 18.7% | 27.5% | 25.0% |
|  |  | No | 86.9% | 73.6% | 82.2% | 81.3% | 72.5% | 75.0% |
| 7 | Not careful as usual | Yes | 11.0% | 23.5% | 16.4% | 18.2% | 27.2% | 22.0% |
|  |  | No | 89.0% | 76.5% | 83.6% | 81.8% | 72.8% | 78.0% |
| 8 | How much did pain interfere | Extremely / Quite a bit | 2.4% | 12.4% | 8.1% | 6.1% | 12.8% | 13.1% |
|  |  | Moderately | 6.1% | 16.2% | 12.7% | 10.5% | 14.2% | 15.1% |
|  |  | A little bit | 34.2% | 28.5% | 29.4% | 32.2% | 31.8% | 28.7% |
|  |  | Not at all | 57.2% | 42.9% | 49.8% | 51.2% | 41.2% | 43.0% |
| 9 | Felt calm and peaceful | None / A little | 2.4% | 8.7% | 6.9% | 11.0% | 17.4% | 11.9% |
|  |  | Some of the time | 14.1% | 23.6% | 17.6% | 30.8% | 31.8% | 32.3% |
|  |  | A good bit of the time | 27.0% | 21.9% | 20.7% | 17.0% | 18.9% | 21.1% |
|  |  | Most of the time | 47.9% | 29.3% | 35.0% | 22.5% | 22.4% | 24.5% |
|  |  | All of the time | 8.7% | 16.4% | 19.8% | 18.6% | 9.5% | 10.2% |
| 10 | Have a lot of energy | None / A little of the time | 5.1% | 14.3% | 10.3% | 11.1% | 23.9% | 22.2% |
|  |  | Some of the time | 23.3% | 29.6% | 26.0% | 32.2% | 36.6% | 38.8% |
|  |  | A good bit of the time | 33.2% | 22.6% | 22.1% | 18.6% | 16.6% | 17.6% |
|  |  | Most of the time | 32.4% | 23.1% | 26.0% | 22.7% | 16.3% | 15.6% |
|  |  | All of the time | 5.9% | 10.5% | 15.6% | 15.5% | 6.6% | 5.8% |
| 11 | Felt downhearted and blue | All / Most | 1.3% | 6.8% | 3.9% | 5.5% | 13.2% | 8.2% |
|  |  | A good bit of the time | 5.5% | 13.4% | 9.5% | 5.6% | 12.6% | 12.6% |
|  |  | Some of the time | 30.1% | 33.0% | 29.1% | 30.1% | 35.5% | 34.0% |
|  |  | A little of the time | 39.1% | 25.1% | 27.2% | 22.1% | 21.6% | 28.0% |
|  |  | None of the time | 24.0% | 21.6% | 30.3% | 36.7% | 17.1% | 17.2% |
| 12 | Health problems interfere with social activities | All / Most | 1.7% | 7.6% | 4.9% | 5.6% | 11.4% | 8.5% |
|  |  | A good bit of the time | 4.1% | 10.7% | 8.3% | 4.9% | 9.1% | 10.0% |
|  |  | Some of the time | 14.9% | 19.6% | 16.8% | 23.0% | 23.2% | 22.8% |
|  |  | A little of the time | 23.0% | 19.7% | 17.8% | 18.8% | 19.4% | 22.4% |
|  |  | None of the time | 56.2% | 42.4% | 52.3% | 47.8% | 36.9% | 36.3% |
