# Supplementary material for: Measurement invariance of the SF-12 among different demographic groups: The HELIUS study
Source: PLoS One. 2018 Sep 13;13(9):e0203483. doi: 10.1371/journal.pone.0203483 (PMC6136718; doi:10.1371/journal.pone.0203483)
Supplement: S3 Table — (DOCX) [file pone.0203483.s003.docx]

**S3 Table. Sensitivity analyses: model fit in separate groups regarding interview mode and language**

|  | **Free parameters^a^** | **χ^2^ (df)** | **RMSEA** | **CFI** |
| --- | --- | --- | --- | --- |
| Mode effect, total sample: |  |  |  |  |
| Interviewer (N=6101) | 51 | 693.787 (45)* | 0.049 (0.045-0.052) | 0.997 |
| Internet (N=9870) | 51 | 956.811 (45)* | 0.045 (0.043-0.048) | 0.993 |
| Paper (N=7175) | 51 | 715.660 (45)* | 0.046 (0.043-0.049) | 0.996 |
| Turks only (N=2766)^a^: |  |  |  |  |
| Turkish language (N=900) | 51 | 160.381 (45)* | 0.053 (0.045-0.062) | 0.994 |
| Dutch language (N=1866) | 51 | 293.161 (45)* | 0.054 (0.049-0.060) | 0.990 |
| Ghanaians only (N=2033)^a^: |  |  |  |  |
| English language (N=1578) | 51 | 259.605 (45)* | 0.055 (0.049-0.062) | 0.992 |
| Dutch language (N=455) | 51 | 140.939 (45)* | 0.068 (0.056-0.081)^b^ | 0.969^b^ |

^a^ We excluded those for which the questionnaire language was uncertain

^b^ Poor model fit (RMSEA>0.055; or CFI<0.97)

*P<0.001
