# Supplementary material for: Measurement invariance of the SF-12 among different demographic groups: The HELIUS study
Source: PLoS One. 2018 Sep 13;13(9):e0203483. doi: 10.1371/journal.pone.0203483 (PMC6136718; doi:10.1371/journal.pone.0203483)
Supplement: S4 Table — (DOCX) [file pone.0203483.s004.docx]

**S4 Table. Sensitivity analyses: measurement invariance analyses regarding interview mode and language**

|  | **Model** | **Free parameters** | **χ^2^(df)** | **RMSEA** | **CFI** |
| --- | --- | --- | --- | --- | --- |
| **Total sample:**  **Interviewer vs. Paper vs. Internet** | 1.Configural | 153 | 2380.453 (135)* | 0.046 (0.045-0.048) | 0.995 |
|  | 2.Metric | 127 | 2909.299 (161)* | 0.047 (0.046-0.049) | 0.994 |
|  | 3.Strong (scalar) | 95 | 4410.018 (193)* | 0.053 (0.052-0.055) | 0.991 |
|  | 4.Strict^a,b^ | 61 | 7027.043 (227)* | 0.062 (0.061-0.064) | 0.986 |
| **Turkish participants:**  **Turkish vs. Dutch language** | 1.Configural | 102 | 457.232 (90)* | 0.054 (0.049-0.059) | 0.992 |
|  | 2.Metric^a^ | 89 | 586.349 (103)* | 0.058 (0.054-0.063) | 0.989 |
|  | 3.Strong (scalar)^a^ | 73 | 814.421 (119)* | 0.065 (0.061-0.069) | 0.985 |
|  | 4.Strict^a^ | 56 | 953.805 (136)* | 0.066 (0.062-0.070) | 0.982 |
| **Ghanaian participants:**  **English vs. Dutch language** | 1.Configural^a^ | 102 | 404.031 (90)* | 0.059 (0.053-0.064) | 0.989 |
|  | 2.Metric^a^ | 89 | 402.835 (103)* | 0.054 (0.048-0.059) | 0.989 |
|  | 3.Strong (scalar)^a,b^ | 73 | 671.258 (119)* | 0.068 (0.063-0.073) | 0.980 |
|  | 4.Strict^a,b^ | 56 | 834.520 (136)* | 0.071 (0.066-0.076) | 0.975 |

* P<0.001

^a^ Poor model fit (RMSEA>0.055; or CFI<0.97)

^b^ Significant worsening of fit compared to previous model (increase in RMSEA>0.05 (metric) or >0.01 (scalar); or decline in CFI>0.004)
