# Supplementary material for: Measurement invariance of the SF-12 among different demographic groups: The HELIUS study
Source: PLoS One. 2018 Sep 13;13(9):e0203483. doi: 10.1371/journal.pone.0203483 (PMC6136718; doi:10.1371/journal.pone.0203483)
Supplement: S5 Table — (DOCX) [file pone.0203483.s005.docx]

**S5 Table. Linear regression with residuals from logistic regression as the outcome and demographic variables as predictors**

| Item nr: | 1 | 2 | 3 | 4 | 5 | 6 | 7 | 8 | 9 | 10 | 11 | 12 |
| --- | --- | --- | --- | --- | --- | --- | --- | --- | --- | --- | --- | --- |
|  | b | b | b | b | b | b | b | b | b | b | b | b |
| Female vs. male | -0.054 | 0.040 | 0.026 | -0.044 | -0.066 | -0.018 | **-0.059** | -0.029 | **0.053** | -0.049 | -0.012 | **-0.038** |
| R^2^ | 0.001 | 0.001 | 0.000 | 0.001 | 0.002 | 0.000 | 0.002 | 0.000 | 0.001 | 0.001 | 0.000 | 0.001 |
| Age 2 vs. 1 | -0,066 | -0,037 | **-0,093** | 0,006 | -0,031 | 0,047 | 0,046 | 0,003 | 0,008 | -0,020 | **-0,062** | -0,026 |
| Age 3 vs. 1 | -0,034 | **-0,062** | -0,051 | **-0,179** | **-0,128** | 0,054 | -0,016 | -0,017 | -0,021 | -0,033 | **-0,058** | -0,050 |
| Age 4 vs. 1 | -0,007 | -0,030 | 0,021 | **-0,254** | **-0,141** | 0,054 | **0,116** | -0,013 | **-0,086** | **-0,116** | **-0,074** | -0,047 |
| Age 5 vs. 1 | -0,023 | 0,051 | **0,219** | **-0,361** | **-0,287** | **0,130** | 0,048 | **-0,129** | **-0,092** | **-0,101** | -0,045 | -0,030 |
| R^2^ | 0.001 | 0.003 | 0.016 | 0.029 | 0.017 | 0.003 | 0.004 | 0.005 | 0.002 | 0.003 | 0.002 | 0.001 |
| Edu mid vs. high | 0.013 | 0.024 | **0.108** | **-0.088** | **-0.089** | -0.004 | -0.032 | 0.006 | 0.045 | -0.017 | 0.033 | -0.028 |
| Edu low vs. high | **0.103** | 0.035 | **0.156** | **-0.332** | **-0.277** | 0.054 | -0.013 | -0.020 | 0.049 | -0.017 | -0.002 | -0.031 |
| R^2^ | 0.003 | 0.000 | 0.006 | 0.024 | 0.021 | 0.001 | 0.000 | 0.000 | 0.001 | 0.000 | 0.001 | 0.001 |
| Sasur vs. NL | **0.141** | **0.054** | -0.005 | **-0.146** | **-0.224** | 0.013 | 0.003 | **0.116** | **0.076** | **-0.056** | **0.098** | **0.066** |
| Afsur vs. NL | **0.112** | **0.033** | **0.115** | **-0.149** | **-0.197** | 0.013 | **0.053** | **0.098** | **0.105** | 0.006 | **0.112** | **0.109** |
| Ghan vs. NL | 0.039 | **0.307** | **0.325** | **-0.237** | **-0.298** | 0.038 | **0.091** | -0.010 | **0.516** | **0.073** | **0.055** | **0.084** |
| Turk vs. NL | **0.110** | **0.124** | **0.086** | **-0.183** | **-0.262** | **-0.199** | **-0.101** | **-0.045** | **0.274** | **0.141** | **0.090** | **-0.018** |
| Mor vs. NL | **0.255** | **0.014** | -0.035 | **-0.181** | **-0.260** | **-0.105** | **-0.121** | **0.048** | **0.255** | **0.251** | **0.056** | 0.004 |
| R^2^ | 0.009 | 0.013 | 0.014 | 0.008 | 0.017 | 0.010 | 0.005 | 0.006 | 0.027 | 0.012 | 0.002 | 0.003 |

Bold coefficients were significant at p<0.05, b=unstandardized regression coefficients, R^2^ = explained variance

Item 1=General Health; Item 2=Limited in moderate activities; Item 3=Limited in climbing several flights; Item 4=Accomplished less physical

Item 5=Limited in work or daily activities; Item 6=Accomplished less emotional ; Item 7=Not careful as usual; Item 8=How much did pain interfere

Item 9=Felt calm and peaceful; Item 10=Have a lot of energy; Item 11=Felt downhearted and blue; Item 12=Health problems interfere with social activities
