# Supplementary material for: Measurement invariance of the SF-12 among different demographic groups: The HELIUS study
Source: PLoS One. 2018 Sep 13;13(9):e0203483. doi: 10.1371/journal.pone.0203483 (PMC6136718; doi:10.1371/journal.pone.0203483)
Supplement: S6 Table — (DOCX) [file pone.0203483.s006.docx]

**S6 Table. Linear regression with residuals from logistic regression as outcome and demographics and demographics*factor scores as predictors**

| Item nr: | 1 | 2 | 3 | 4 | 5 | 6 | 7 | 8 | 9 | 10 | 11 | 12 |
| --- | --- | --- | --- | --- | --- | --- | --- | --- | --- | --- | --- | --- |
|  | b | b | b | b | b | b | b | b | b | b | b | b |
| Female vs. male*physical factor score | -0.012 | -0.019 | **-0.017** | -0.002 | -0.006 |  |  | **-0.014** |  | -0.006 |  | -0.014 |
| Female vs. male*mental factor score | 0.004 |  |  |  |  | 0.001 | 0.000 |  | **-0.019** | -0.017 | -0.008 | 0.011 |
| R^2^ | 0.002 | 0.003 | 0.001 | 0.001 | 0.002 | 0.000 | 0.002 | 0.001 | 0.002 | 0.002 | 0.000 | 0.002 |
| Age 2 vs. 1*physical factor score | -0.024 | 0.006 | **0.100** | **-0.238** | **-0.186** |  |  | **-0.112** |  | -0.042 |  | **-0.112** |
| Age 3 vs. 1*physical factor score | 0.003 | 0.026 | 0.038 | **-0.048** | -0.028 |  |  | **-0.043** |  | -0.056 |  | -0.015 |
| Age 4 vs. 1*physical factor score | -0.040 | -0.004 | -0.023 | 0.020 | -0.015 |  |  | -0.020 |  | -0.018 |  | -0.020 |
| Age 5 vs. 1*physical factor score | 0.004 | **-0.070** | **-0.074** | **0.058** | **0.064** |  |  | **0.053** |  | -0.052 |  | -0.026 |
| Age 2 vs. 1*mental factor score | 0.058 |  |  |  |  | -0.012 | -0.043 |  | -0.023 | -0.052 | -0.011 | **0.090** |
| Age 3 vs. 1*mental factor score | -0.016 |  |  |  |  | 0.015 | 0.036 |  | -0.042 | 0.009 | -0.001 | -0.001 |
| Age 4 vs. 1*mental factor score | 0.016 |  |  |  |  | -0.010 | -0.039 |  | 0.005 | 0.051 | 0.024 | 0.035 |
| Age 5 vs. 1*mental factor score | -0.020 |  |  |  |  | **-0.061** | -0.003 |  | 0.008 | **0.074** | -0.012 | 0.026 |
| R^2^ | 0.002 | 0.008 | 0.022 | 0.043 | 0.028 | 0.004 | 0.006 | 0.012 | 0.003 | 0.007 | 0.003 | 0.005 |
| Edu mid vs. high*physical factor score | -0.010 | -0.044 | **-0.072** | -0.013 | -0.038 |  |  | -0.048 |  | -0.024 |  | -0.049 |
| Edu low vs. high*physical factor score | -0.020 | **-0.059** | -0.043 | **0.098** | 0.029 |  |  | 0.018 |  | **-0.099** |  | 0.000 |
| Edu mid vs. high*mental factor score | -0.018 |  |  |  |  | 0.046 | 0.028 |  | -0.019 | -0.005 | **-0.050** | 0.054 |
| Edu low vs. high*mental factor score | 0.003 |  |  |  |  | -0.022 | 0.037 |  | -0.065 | **0.095** | 0.001 | 0.025 |
| R^2^ | 0.003 | 0.002 | 0.008 | 0.026 | 0.022 | 0.001 | 0.001 | 0.001 | 0.002 | 0.001 | 0.002 | 0.002 |
| Sasur vs. NL*physical factor score | **-0.102** | -0.027 | -0.015 | -0.002 | 0.006 |  |  | **-0.077** |  | 0.011 |  | -0.013 |
| Afsur vs. NL*physical factor score | **-0.094** | -0.012 | **-0.041** | **-0.050** | **-0.047** |  |  | **-0.075** |  | 0.013 |  | -0.031 |
| Ghan vs. NL*physical factor score | **0.103** | **-0.153** | **-0.190** | 0.025 | **0.063** |  |  | 0.017 |  | **-0.087** |  | **0.085** |
| Turk vs. NL*physical factor score | **-0.076** | **-0.032** | -0.025 | -0.026 | -0.003 |  |  | 0.007 |  | 0.006 |  | **0.154** |
| Mor vs. NL*physical factor score | **-0.152** | -0.005 | -0.009 | 0.011 | 0.005 |  |  | **-0.046** |  | 0.002 |  | 0.025 |
| Sasur vs. NL*mental factor score | **0.089** |  |  |  |  | **-0.034** | 0.001 |  | 0.029 | -0.001 | **-0.036** | -0.027 |
| Afsur vs. NL*mental factor score | **0.076** |  |  |  |  | **-0.051** | **-0.040** |  | 0.027 | -0.008 | **-0.039** | -0.038 |
| Ghan vs. NL*mental factor score | 0.017 |  |  |  |  | **-0.098** | **-0.134** |  | **0.077** | **0.151** | 0.007 | -0.039 |
| Turk vs. NL*mental factor score | **0.103** |  |  |  |  | **0.103** | **0.037** |  | **-0.042** | 0.032 | -0.015 | **-0.133** |
| Mor vs. NL*mental factor score | **0.100** |  |  |  |  | 0.014 | **0.059** |  | **-0.077** | 0.026 | -0.027 | **-0.064** |
| R^2^ | 0.014 | 0.015 | 0.018 | 0.009 | 0.018 | 0.014 | 0.008 | 0.009 | 0.029 | 0.013 | 0.003 | 0.007 |

Bold coefficients were significant at p<0.05, b=unstandardized regression coefficients, R^2^ = explained variance

Item 1=General Health; Item 2=Limited in moderate activities; Item 3=Limited in climbing several flights; Item 4=Accomplished less physical

Item 5=Limited in work or daily activities; Item 6=Accomplished less emotional ; Item 7=Not careful as usual ; Item 8=How much did pain interfere

Item 9=Felt calm and peaceful; Item 10=Have a lot of energy; Item 11=Felt downhearted and blue; Item 12=Health problems interfere with social activities
