# Supplementary material for: Measurement invariance of the SF-12 among different demographic groups: The HELIUS study
Source: PLoS One. 2018 Sep 13;13(9):e0203483. doi: 10.1371/journal.pone.0203483 (PMC6136718; doi:10.1371/journal.pone.0203483)
Supplement: S7 Table — (DOCX) [file pone.0203483.s007.docx]

**S7 Table.**

**Factor means and variances*, and standardized mean differences, in strict and partial strict multiple group confirmatory factor models**

|  | **Physical health** | | | | | | | | | | **Mental health** | | | | | | | | | | | |
| --- | --- | --- | --- | --- | --- | --- | --- | --- | --- | --- | --- | --- | --- | --- | --- | --- | --- | --- | --- | --- | --- | --- |
|  | **Not DIF adjusted** | | | | | **DIF adjusted** | | | | | **Not DIF adjusted** | | | | | | **DIF adjusted** | | | | | |
|  |  |  |  | 95% CI | |  |  |  | 95% CI | |  |  |  | 95% CI | |  | |  |  | 95% CI | |  |
| **Dutch origin sample** | Mean | Variance | d | lower | upper | Mean | Variance | d | lower | upper | Mean | Variance | d | lower | upper | Mean | | Variance | d | lower | upper |  |
| 18-30 years (ref) | 0 | 1 |  |  |  | 0 | 1 |  |  |  | 0 | 1 |  |  |  | 0 | | 1 |  |  |  |  |
| 31-40 years | -0.069 | 1.140 | -0.07 | -0.16 | 0.03 | -0.083 | 1.156 | -0.08 | -0.18 | 0.02 | 0.124 | 1.082 | 0.12 | 0.03 | 0.22 | 0.126 | | 1.083 | 0.12 | 0.03 | 0.22 |  |
| 41-50 years | -0.292 | 2.016 | -0.24 | -0.33 | -0.15 | -0.391 | 2.138 | -0.31 | -0.40 | -0.22 | 0.291 | 1.682 | 0.25 | 0.16 | 0.34 | 0.309 | | 1.684 | 0.27 | 0.17 | 0.36 |  |
| 51-60 years | -0.546 | 1.902 | -0.45 | -0.54 | -0.36 | -0.656 | 1.998 | -0.54 | -0.63 | -0.45 | 0.416 | 1.642 | 0.36 | 0.27 | 0.45 | 0.434 | | 1.643 | 0.38 | 0.29 | 0.47 |  |
| 61-70 years | -0.667 | 2.260 | -0.52 | -0.62 | -0.43 | -0.806 | 2.379 | -0.62 | -0.72 | -0.52 | 0.795 | 1.639 | 0.69 | 0.60 | 0.79 | 0.817 | | 1.642 | 0.711 | 0.614 | 0.81 |  |
|  |  |  |  |  |  |  |  |  |  |  |  |  |  |  |  |  | |  |  |  |  |  |
| High education (ref) | 0 | 1 |  |  |  | 0 | 1 |  |  |  | 0 | 1 |  |  |  | 0 | | 1 |  |  |  |  |
| Medium education | -0.312 | 1.023 | -0.31 | -0.38 | -0.24 | -0.362 | 1.044 | -0.36 | -0.43 | -0.29 | -0.086 | 1.241 | -0.08 | -0.15 | -0.01 | -0.074 | | 1.246 | -0.07 | -0.14 | 0.00 |  |
| Low education | -0.810 | 1.321 | -0.75 | -0.83 | -0.67 | -0.965 | 1.352 | -0.89 | -0.97 | -0.81 | 0.063 | 1.628 | 0.06 | -0.02 | 0.13 | 0.097 | | 1.644 | 0.08 | 0.01 | 0.16 |  |
|  |  |  |  |  |  |  |  |  |  |  |  |  |  |  |  |  | |  |  |  |  |  |
| **Total sample** |  |  |  |  |  |  |  |  |  |  |  |  |  |  |  |  | |  |  |  |  |  |
| Dutch (ref) | 0 | 1 |  |  |  | 0 | 1 |  |  |  | 0 | 1 |  |  |  | 0 | | 1 |  |  |  |  |
| South-Asian Surinamese | -0.675 | 1.100 | -0.66 | -0.70 | -0.61 | -0.601 | 1.464 | -0.54 | -0.59 | -0.50 | -0.406 | 1.205 | -0.39 | -0.43 | -0.34 | -0.487 | | 1.166 | -0.47 | -0.51 | -0.42 |  |
| African Surinamese | -0.412 | 1.073 | -0.40 | -0.45 | -0.36 | -0.283 | 1.520 | -0.25 | -0.29 | -0.21 | -0.078 | 1.196 | -0.07 | -0.12 | -0.03 | -0.134 | | 1.25 | -0.13 | -0.17 | -0.09 |  |
| Ghanaian | -0.558 | 0.723 | -0.60 | -0.65 | -0.55 | -0.296 | 1.024 | -0.29 | -0.34 | -0.24 | -0.253 | 0.791 | -0.27 | -0.32 | -0.22 | -0.142 | | 1.233 | -0.13 | -0.18 | -0.09 |  |
| Turkish | -0.836 | 1.135 | -0.81 | -0.85 | -0.77 | -0.637 | 1.539 | -0.57 | -0.61 | -0.52 | -0.763 | 1.022 | -0.76 | -0.80 | -0.72 | -0.726 | | 1.015 | -0.72 | -0.77 | -0.68 |  |
| Moroccan | -0.744 | 1.198 | -0.71 | -0.75 | -0.67 | -0.613 | 1.549 | -0.54 | -0.59 | -0.50 | -0.636 | 1.007 | -0.63 | -0.68 | -0.59 | -0.572 | | 1.047 | -0.57 | -0.61 | -0.52 |  |

*Means and variances come from different models (strict vs partial strict model per demographic variable) and thus can only be compared within one demographic characteristic (gender, age, education or ethnicity).
