# Supplementary material for: Measurement invariance of the SF-12 among different demographic groups: The HELIUS study
Source: PLoS One. 2018 Sep 13;13(9):e0203483. doi: 10.1371/journal.pone.0203483 (PMC6136718; doi:10.1371/journal.pone.0203483)
Supplement: S8 Table — (DOCX) [file pone.0203483.s008.docx]

**S8 Table. Unstandardized results from partial strict model for ethnicity**

|  | | **Description** | | **All** | | **Dutch** | | **South-Asian Surinamese** | | **African Surinamese** | | **Ghanaian** | | **Turkish** | | **Moroccan** | |
| --- | --- | --- | --- | --- | --- | --- | --- | --- | --- | --- | --- | --- | --- | --- | --- | --- | --- |
| Factor loadings | | |  |  |  | |  | |  | |  | |  | |  | |  |
| Physical | #1 | General health | |  | | 1.041 | | 0.825 | | 0.804 | | 0.653 | | 0.984 | | 1.080 | |
|  | #2 | Limited moderate activities | |  | | 1.663 | | 1.322 | | 1.228 | | 0.732 | | 1.183 | | 1.212 | |
|  | #3 | Limited in climbing flights | |  | | 1.188 | | 1.074 | | 0.956 | | 0.767 | | 0.988 | | 1.069 | |
|  | #4 | Accomplished less physical | | 1.359 | |  | |  | |  | |  | |  | |  | |
|  | #5 | Limited in work/daily activities | |  | | 1.645 | | 1.491 | | 1.769 | | 1.436 | | 1.610 | | 1.460 | |
|  | #8 | Pain | | 1.389 | |  | |  | |  | |  | |  | |  | |
|  | #10 | Energy | |  | | 0.526 | | 0.412 | | 0.359 | | 0.694 | | 0.526 | | 0.531 | |
|  | #12 | Social activities | | 0.378 | |  | |  | |  | |  | |  | |  | |
| Mental | #1 | General Health | |  | | 0.218 | | 0.134 | | 0.094 | | 0.006 | | -0.036 | | 0.087 | |
|  | #6 | Accomplished less emotional | |  | | 2.545 | | 1.711 | | 1.792 | | 1.580 | | 1.498 | | 1.762 | |
|  | #7 | Not as careful as usual | | 1.580 | |  | |  | |  | |  | |  | | 1.580 | |
|  | #9 | Calm and peaceful | |  | | 0.863 | | 0.865 | | 0.746 | | 0.327 | | 0.996 | | 0.951 | |
|  | #10 | Energy | |  | | 0.687 | | 0.536 | | 0.509 | | -0.104 | | 0.301 | | 0.323 | |
|  | #11 | Downhearted and blue | | 1.246 | |  | |  | |  | |  | |  | |  | |
|  | #12 | Social activities | | 0.969 | |  | |  | |  | |  | |  | |  | |
| Physical with mental | |  | |  | | 0.440 | | 0.987 | | 0.954 | | 0.815 | | 0.950 | | 0.915 | |
| #2 with # 3 | |  | |  | | 0.582 | | 0.562 | | 0.636 | | 0.787 | | 0.570 | | 0.601 | |
| #4 with #5 | |  | |  | | 0.777 | | 0.799 | | 0.885 | | 0.821 | | 0.758 | | 0.746 | |
| #6 with #7 | |  | |  | | 0.730 | | 0.847 | | 0.812 | | 0.816 | | 0.827 | | 0.815 | |
| #9 with #10 | |  | |  | | 0.252 | | 0.273 | | 0.355 | | 0.528 | | 0.434 | | 0.379 | |
| #9 with #11 | |  | |  | | 0.247 | | 0.057 | | 0.051 | | -0.243 | | -0.178 | | -0.081 | |
|  | |  | |  | |  | |  | |  | |  | |  | |  | |
| Mean physical | |  | |  | | 0 | | -0.602 | | -0.284 | | -0.299 | | -0.632 | | -0.610 | |
| Mean mental | |  | |  | | 0 | | -0.483 | | -0.127 | | -0.124 | | -0.754 | | -0.588 | |
| Variance Physical | |  | |  | | 1 | | 1.449 | | 1.506 | | 1.010 | | 1.518 | | 1.534 | |
| Variance Mental | |  | |  | | 1 | | 1.221 | | 1.311 | | 1.316 | | 1.113 | | 1.110 | |
|  | |  | |  | |  | |  | |  | |  | |  | |  | |
| Thresholds | |  | |  | |  | |  | |  | |  | |  | |  | |
| # 1 | $1 | Good (vs. fair/poor) | |  | | -1.993 | | -1.305 | | -1.351 | | -1.220 | | -1.240 | | -1.284 | |
|  | $2 | Very good | |  | | 0.300 | | 0.903 | | 0.916 | | 0.445 | | 1.024 | | 1.125 | |
|  | $3 | Excellent | |  | | 1.802 | | 1.791 | | 1.843 | | 1.328 | | 2.153 | | 2.215 | |
| #2 | $1 | Yes, limited a little (vs. yes a lot) | |  | | -4.398 | | -3.700 | | -3.697 | | -1.766 | | -2.897 | | -3.459 | |
|  | $2 | No, not limited at all | |  | | -2.256 | | -1.595 | | -1.616 | | -0.620 | | -1.135 | | -1.479 | |
| #3 | $1 | Yes, limited a little (vs. yes a lot) | |  | | -3.498 | | -3.213 | | -3.016 | | -1.652 | | -2.505 | | -3.094 | |
|  | $2 | No, not limited at all | |  | | -1.610 | | -1.320 | | -1.075 | | -0.490 | | -0.919 | | -1.304 | |
| #4 | $1 | No (vs. yes) | | -1.668 | |  | |  | |  | |  | |  | |  | |
| #5 | $1 | No (vs. yes) | |  | | -1.689 | | -1.942 | | -2.270 | | -1.922 | | -1.939 | | -1.932 | |
| #6 | $1 | No (vs. yes) | |  | | -3.066 | | -2.178 | | -2.332 | | -1.696 | | -2.250 | | -2.457 | |
| #7 | $1 | No (vs. yes) | | -2.294 | |  | |  | |  | |  | |  | |  | |
| #8 | $1 | Moderately | | -3.139 | |  | |  | |  | |  | |  | |  | |
|  | $2 | A little bit | | -2.071 | |  | |  | |  | |  | |  | |  | |
|  | $3 | Not at all | | -0.419 | |  | |  | |  | |  | |  | |  | |
| #9 | $1 | Some of the time (vs. extremely/quite a bit) | |  | | -2.619 | | -2.297 | | -2.047 | | -1.350 | | -2.112 | | -2.226 | |
|  | $2 | A good bit of the time | |  | | -1.287 | | -1.053 | | -1.002 | | -0.261 | | -0.780 | | -0.764 | |
|  | $3 | Most of the time | |  | | -0.218 | | -0.271 | | -0.252 | | 0.199 | | -0.068 | | 0.000 | |
|  | $4 | All of the time | |  | | 1.795 | | 0.933 | | 1.020 | | 0.912 | | 1.147 | | 1.239 | |
| #10 | $1 | Some of the time (vs. none / a little) | |  | | -2.352 | | -2.029 | | -1.903 | | -1.633 | | -1.517 | | -1.556 | |
|  | $2 | A good bit of the time | |  | | -0.821 | | -0.725 | | -0.648 | | -0.394 | | -0.200 | | -0.130 | |
|  | $3 | Most of the time | |  | | 0.425 | | 0.100 | | 0.124 | | 0.159 | | 0.442 | | 0.571 | |
|  | $4 | All of the time | |  | | 2.243 | | 1.284 | | 1.221 | | 0.999 | | 1.475 | | 1.635 | |
| #11 | $1 | A good bit of the time (vs. all / most) | | -3.037 | |  | |  | |  | |  | |  | |  | |
|  | $2 | Some of the time | | -2.113 | |  | |  | |  | |  | |  | |  | |
|  | $3 | A little of the time | | -0.504 | |  | |  | |  | |  | |  | |  | |
|  | $4 | None of the time | | 0.790 | |  | |  | |  | |  | |  | |  | |
| #12 | $1 | A good bit of the time (vs. all /most) | | -3.131 | |  | |  | |  | |  | |  | |  | |
|  | $2 | Some of the time | | -2.337 | |  | |  | |  | |  | |  | |  | |
|  | $3 | A little of the time | | -1.192 | |  | |  | |  | |  | |  | |  | |
|  | $4 | None of the time | | -0.282 | |  | |  | |  | |  | |  | |  | |
|  | |  | |  | |  | |  | |  | |  | |  | |  | |
| Residual variances all items | |  | | 1 | |  | |  | |  | |  | |  | |  | |
