# Supplementary material for: Measurement invariance of the SF-12 among different demographic groups: The HELIUS study
Source: PLoS One. 2018 Sep 13;13(9):e0203483. doi: 10.1371/journal.pone.0203483 (PMC6136718; doi:10.1371/journal.pone.0203483)
Supplement: S9 Table — (DOCX) [file pone.0203483.s009.docx]

**S9 Table. Standardized results (StdYX) – from partial strict model for ethnicity**

|  | | **Description** | | **All** | | **Dutch** | **South-Asian Surinamese** | | **African Surinamese** | | **Ghanaian** | | **Turkish** | | **Moroccan** | |  |
| --- | --- | --- | --- | --- | --- | --- | --- | --- | --- | --- | --- | --- | --- | --- | --- | --- | --- |
| Factor loadings | | |  | |  |  | |  | |  | |  | |  | |  | |
| Physical | #1 | General health | |  | | 0.682 | 0.666 | | 0.676 | | 0.547 | | 0.782 | | 0.777 | |  |
|  | #2 | Limited moderate activities | |  | | 0.857 | 0.847 | | 0.833 | | 0.593 | | 0.825 | | 0.832 | |  |
|  | #3 | Limited in climbing flights | |  | | 0.765 | 0.791 | | 0.761 | | 0.610 | | 0.773 | | 0.798 | |  |
|  | #4 | Accomplished less physical | |  | | 0.805 | 0.853 | | 0.858 | | 0.807 | | 0.859 | | 0.860 | |  |
|  | #5 | Limited in work/daily activities | |  | | 0.855 | 0.874 | | 0.908 | | 0.822 | | 0.893 | | 0.875 | |  |
|  | #8 | Pain | |  | | 0.812 | 0.858 | | 0.863 | | 0.813 | | 0.863 | | 0.865 | |  |
|  | #10 | Energy | |  | | 0.366 | 0.348 | | 0.321 | | 0.593 | | 0.480 | | 0.482 | |  |
|  | #12 | Social activities | |  | | 0.244 | 0.260 | | 0.262 | | 0.220 | | 0.271 | | 0.274 | |  |
| Mental | #1 | General Health | |  | | 0.143 | 0.099 | | 0.074 | | 0.006 | | -0.024 | | 0.052 | |  |
|  | #6 | Accomplished less emotional | |  | | 0.931 | 0.884 | | 0.899 | | 0.866 | | 0.845 | | 0.880 | |  |
|  | #7 | Not as careful as usual | |  | | 0.845 | 0.868 | | 0.875 | | 0.876 | | 0.858 | | 0.857 | |  |
|  | #9 | Calm and peaceful | |  | | 0.653 | 0.691 | | 0.649 | | 0.351 | | 0.724 | | 0.708 | |  |
|  | #10 | Energy | |  | | 0.478 | 0.415 | | 0.425 | | -0.101 | | 0.235 | | 0.249 | |  |
|  | #11 | Downhearted and blue | |  | | 0.780 | 0.809 | | 0.819 | | 0.819 | | 0.796 | | 0.795 | |  |
|  | #12 | Social activities | |  | | 0.625 | 0.610 | | 0.626 | | 0.644 | | 0.594 | | 0.596 | |  |
| Physical with mental | |  | |  | | 0.440 | 0.742 | | 0.679 | | 0.707 | | 0.731 | | 0.701 | |  |
| #2 with # 3 | |  | |  | | 0.582 | 0.562 | | 0.636 | | 0.787 | | 0.570 | | 0.601 | |  |
| #4 with #5 | |  | |  | | 0.777 | 0.799 | | 0.885 | | 0.821 | | 0.758 | | 0.746 | |  |
| #6 with #7 | |  | |  | | 0.730 | 0.847 | | 0.812 | | 0.816 | | 0.827 | | 0.815 | |  |
| #9 with #10 | |  | |  | | 0.252 | 0.273 | | 0.355 | | 0.528 | | 0.434 | | 0.379 | |  |
| #9 with #11 | |  | |  | | 0.247 | 0.057 | | 0.051 | | -0.243 | | -0.178 | | -0.081 | |  |
|  | |  | |  | |  |  | |  | |  | |  | |  | |  |
| Mean physical | |  | |  | | 0 | -0.500 | | -0.231 | | -0.298 | | -0.513 | | -0.493 | |  |
| Mean mental | |  | |  | | 0 | -0.437 | | -0.111 | | -0.108 | | -0.715 | | -0.558 | |  |
| Variance Physical | |  | |  | | 1 | 1 | | 1 | | 1 | | 1 | | 1 | |  |
| Variance Mental | |  | |  | | 1 | 1 | | 1 | | 1 | | 1 | | 1 | |  |
|  | |  | |  | |  |  | |  | |  | |  | |  | |  |
| Thresholds | |  | |  | |  |  | |  | |  | |  | |  | |  |
| # 1 | $1 | Good (vs. fair/poor) | |  | | -1.305 | -0.874 | | -0.926 | | -1.018 | | -0.800 | | -0.746 | |  |
|  | $2 | Very good | |  | | 0.197 | 0.605 | | 0.628 | | 0.371 | | 0.660 | | 0.654 | |  |
|  | $3 | Excellent | |  | | 1.180 | 1.200 | | 1.264 | | 1.108 | | 1.389 | | 1.287 | |  |
| #2 | $1 | Yes, limited a little (vs. yes a lot) | |  | | -2.267 | -1.969 | | -2.044 | | -1.422 | | -1.639 | | -1.918 | |  |
|  | $2 | No, not limited at all | |  | | -1.163 | -0.849 | | -0.893 | | -0.499 | | -0.642 | | -0.820 | |  |
| #3 | $1 | Yes, limited a little (vs. yes a lot) | |  | | -2.253 | -1.966 | | -1.956 | | -1.309 | | -1.591 | | -1.865 | |  |
|  | $2 | No, not limited at all | |  | | -1.037 | -0.808 | | -0.697 | | -0.388 | | -0.583 | | -0.786 | |  |
| #4 | $1 | No (vs. yes) | |  | | -0.989 | -0.870 | | -0.858 | | -0.986 | | -0.855 | | -0.852 | |  |
| #5 | $1 | No (vs. yes) | |  | | -0.877 | -0.945 | | -0.950 | | -1.095 | | -0.873 | | -0.935 | |  |
| #6 | $1 | No (vs. yes) | |  | | -1.121 | -1.018 | | -1.022 | | -0.984 | | -1.203 | | -1.165 | |  |
| #7 | $1 | No (vs. yes) | |  | | -1.227 | -1.140 | | -1.110 | | -1.108 | | -1.180 | | -1.181 | |  |
| #8 | $1 | Moderately | |  | | -1.833 | -1.611 | | -1.588 | | -1.828 | | -1.583 | | -1.577 | |  |
|  | $2 | A little bit | |  | | -1.210 | -1.063 | | -1.048 | | -1.206 | | -1.045 | | -1.041 | |  |
|  | $3 | Not at all | |  | | -0.245 | -0.215 | | -0.212 | | -0.244 | | -0.211 | | -0.211 | |  |
| #9 | $1 | Some of the time (vs. extremely/quite a bit) | |  | | -1.983 | -1.660 | | -1.557 | | -1.264 | | -1.456 | | -1.573 | |  |
|  | $2 | A good bit of the time | |  | | -0.974 | -0.761 | | -0.762 | | -0.244 | | -0.538 | | -0.540 | |  |
|  | $3 | Most of the time | |  | | -0.165 | -0.196 | | -0.192 | | 0.186 | | -0.047 | | 0.000 | |  |
|  | $4 | All of the time | |  | | 1.359 | 0.674 | | 0.776 | | 0.854 | | 0.790 | | 0.875 | |  |
| #10 | $1 | Some of the time (vs. none / a little) | |  | | -1.636 | -1.423 | | -1.387 | | -1.389 | | -1.124 | | -1.141 | |  |
|  | $2 | A good bit of the time | |  | | -0.571 | -0.509 | | -0.472 | | -0.335 | | -0.148 | | -0.095 | |  |
|  | $3 | Most of the time | |  | | 0.296 | 0.070 | | 0.090 | | 0.135 | | 0.328 | | 0.419 | |  |
|  | $4 | All of the time | |  | | 1.560 | 0.903 | | 0.890 | | 0.849 | | 1.092 | | 1.199 | |  |
| #11 | $1 | A good bit of the time (vs. all / most) | |  | | -1.901 | -1.785 | | -1.743 | | -1.741 | | -1.839 | | -1.840 | |  |
|  | $2 | Some of the time | |  | | -1.323 | -1.242 | | -1.213 | | -1.211 | | -1.279 | | -1.280 | |  |
|  | $3 | A little of the time | |  | | -0.315 | -0.296 | | -0.289 | | -0.289 | | -0.305 | | -0.305 | |  |
|  | $4 | None of the time | |  | | 0.495 | 0.464 | | 0.454 | | 0.453 | | 0.478 | | 0.479 | |  |
| #12 | $1 | A good bit of the time (vs. all /most) | |  | | -2.019 | -1.785 | | -1.765 | | -1.814 | | -1.820 | | -1.828 | |  |
|  | $2 | Some of the time | |  | | -1.507 | -1.332 | | -1.318 | | -1.354 | | -1.359 | | -1.365 | |  |
|  | $3 | A little of the time | |  | | -0.769 | -0.679 | | -0.672 | | -0.691 | | -0.693 | | -0.696 | |  |
|  | $4 | None of the time | |  | | -0.182 | -0.161 | | -0.159 | | -0.163 | | -0.164 | | -0.164 | |  |
|  | |  | |  | |  |  | |  | |  | |  | |  | |  |
| Residual variances | | #1 | |  | | 0.429 | 0.449 | | 0.470 | | 0.696 | | 0.416 | | 0.337 | |  |
|  | | #2 | |  | | 0.266 | 0.283 | | 0.306 | | 0.649 | | 0.320 | | 0.308 | |  |
|  | | #3 | |  | | 0.415 | 0.374 | | 0.421 | | 0.628 | | 0.403 | | 0.363 | |  |
|  | | #4 | |  | | 0.351 | 0.272 | | 0.264 | | 0.349 | | 0.263 | | 0.261 | |  |
|  | | #5 | |  | | 0.270 | 0.237 | | 0.175 | | 0.325 | | 0.203 | | 0.234 | |  |
|  | | #6 | |  | | 0.134 | 0.219 | | 0.192 | | 0.250 | | 0.286 | | 0.225 | |  |
|  | | #7 | |  | | 0.286 | 0.247 | | 0.234 | | 0.233 | | 0.265 | | 0.265 | |  |
|  | | #8 | |  | | 0.341 | 0.263 | | 0.256 | | 0.339 | | 0.254 | | 0.252 | |  |
|  | | #9 | |  | | 0.573 | 0.522 | | 0.579 | | 0.876 | | 0.475 | | 0.499 | |  |
|  | | #10 | |  | | 0.484 | 0.492 | | 0.531 | | 0.723 | | 0.549 | | 0.537 | |  |
|  | | #11 | |  | | 0.392 | 0.345 | | 0.330 | | 0.329 | | 0.367 | | 0.367 | |  |
|  | | #12 | |  | | 0.416 | 0.325 | | 0.318 | | 0.336 | | 0.338 | | 0.341 | |  |
